# Gender, Socioeconomic Status, Cultural Differences, Education, Family Size and Procrastination: A Sociodemographic Meta-Analysis

**DOI:** 10.3389/fpsyg.2021.719425

**Published:** 2022-01-05

**Authors:** Desheng Lu, Yiheng He, Yu Tan

**Affiliations:** ^1^Department of Educational Science, Sichuan Normal University, Chengdu, China; ^2^Institute of Multicultural Science, Sichuan Normal University, Chengdu, China

**Keywords:** procrastination, sociodemographics, multicultures, meta-analysis, gender

## Abstract

Procrastination describes a ubiquitous scenario in which individuals voluntarily postpone scheduled activities at the expense of adverse consequences. Steel (2007) pioneered a meta-analysis to explicitly reveal the nature of procrastination and sparked intensive research on its demographic characteristics. However, conflicting and heterogeneous findings reported in the existing literature make it difficult to draw reliable conclusions. In addition, there is still room to further investigate on more sociodemographic features that include socioeconomic status, cultural differences and procrastination education. To this end, we performed quantitative sociodemographic meta-analyses (*k* = 193, total *n* = 106,764) to fill this gap. It was found that the general tendency and academic procrastination tendency of males were stronger than females (*r* = 0.04, 95% CI: 0.02–0.05). No significant effects of differences in socioeconomic status (i.e., poor or rich), multiculturalism (i.e., Han nation or minorities), nationality (i.e., China or other countries), family size (i.e., one child or > 1 child), and educational background (i.e., science or arts/literature) were found to affect procrastination tendencies. Furthermore, it was noteworthy that the gender differences in procrastination tendencies were prominently moderated by measurements, which has a greater effect on the Aitken Procrastination Inventory (API) (*r* = 0.035, 95% CI: −0.01–0.08) than on the General Procrastination Scale (GPS) (*r* = 0.018, 95% CI: −0.01–0.05). In conclusion, this study provides robust evidence that males tended to procrastinate more than females in general and academic profiles, and further indicates that procrastination tendencies do not vary based on sociodemographic situations, including socioeconomic status, multiculturalism, nationality, family size, and educational background.

## Introduction

Procrastination is a stable harmful tendency within individuals, defined as the voluntary but irrational delay of intended course of actions (Elliot, 2002; Steel, 2007). In addition, the procrastination also refers to an off behavior that keeps unnecessary delay and reap negative consequences caused by this delay *per se* (van Eerde, 2000, 2003). The absolute number of procrastinators is sizeable (Potts, 1987), with approximately 75% of college students considering themselves procrastinators and nearly half of them procrastinating consistently and problematically (Steel and Ferrari, 2013). Furthermore, procrastination significantly harms people’s health, well-being, work efficiency and academic performance (Kachgal et al., 2001; Sirois, 2007; Balkis and Erdinç, 2017). An earlier meta-analysis thoroughly and explicitly reviewed the nature of procrastination and revealed how procrastination is associated with many variables, such as personality, mental health and demographic features (van Eerde, 2003; Eerde, 2004; Steel, 2007).

However, the conflicting results for the association between gender and procrastination were observed frequently in existing studies. For instance, Li (2013) and colleagues used large-scale sample to report the strong effect for the gender differences in procrastination among Chinese students, with more procrastination in males (Li, 2013). In addition, this conclusion is also supported by the Turkish population (Nilufer, 2017). However, the inconsistent results reported show that there is no gender differences in procrastination (Ajayi, 2020; Wang, 2020). To make matters worse, a portion of studies provided evidence to claim more procrastination in females instead of males (Bian, 2017; Song et al., 2020). In this vein, so far there is no solid evidence to clarify this association. Furthermore, as the close linkage between socioeconomic status (SES) and the self-regulation (Miller et al., 2015), the role of SES on procrastination that caused by the failure of self-regulation also caught our eyes. Nevertheless, results for such relations were found heterogeneous: Yao (2020) reported a significant negative correlation between SES and procrastination (Yao, 2020), whereas provided null findings in other studies (Huang et al., 2017; Xing, 2019). Thus, the needs for a meta-analytic evidence to clarify the association between SES and procrastination emerged. Further, the association between education and procrastination is still sparked much interests for us, but remains inconsistent conclusions. Ferrari et al. (2009a) have demonstrated results for claiming the negative relationship between education and procrastination (Ferrari et al., 2009a). However, graduates students were found procrastinate more than students in high and middle schools (Wen, 2014; Li, 2019). Thus, it leads us to infer the association between the education and procrastination, as well to promote a need to clarify what the direction is for such influence.

A considerable body of meta-analyses on procrastination has been conducted. Steel (2007) provided the correlation between procrastination and other psychological features, and revealed that task aversiveness, task delay, self-efficacy, impulsiveness, conscientiousness, self-control, distractibility, organization, and achievement motivation are strong predictors of procrastination. However, demographic features such as age are not significant predictors of procrastination (Steel, 2007). More recent meta-analyses have discussed demographic features of procrastination (Balkis and Erdinç, 2017; Krispenz et al., 2019) and focused on the relation between specific topics such as procrastination and time perspective (Sirois, 2014), academic performance (Kim and Seo, 2015; Sæle et al., 2017) and intervention (van Eerde and Klingsieck, 2018). In addition to the general demographic features of procrastination and these specific features, other social factors should be probed, including SES, cultural differences, and educational background.

Both 4-dimensional theoretical model and competing theory of SES have suggested that SES is defined as a measure of individuals’ combined economic and social status, which may influence many aspects of personal behaviors, such as smoking, addiction and drinking (Pampel and Rogers, 2004; Cutler et al., 2008; Baker, 2014). A previous study suggested that individuals with lower SES procrastinate in treatment or hospitalization and/or do have apply for health insurance (Weissman et al., 1991). In addition, lower SES affects the procrastination tendency of college students, as students with lower SES are more worried about their financial situation, which triggers anxiety and leads to increased academic procrastination (Stöber and Joormann, 2001). Similarly, differences in SES can also explain to certain extent the tendency of individuals to use Facebook to avoid or procrastinate on tasks (Arnett, 2016). Furthermore, a recent study measured childhood SES and investigated its relationship with procrastination found that SES is closely related to procrastination *via* parenting style and conscientiousness trait (Shimamura et al., 2021). Such associations could be attributed to the personality-trait formation. In detailed, the high SES was found to make children prone to form conscientiousness trait that is significantly factor to persist procrastination (Noftle and Robins, 2007; O’Connor and Paunonen, 2007; Lubbers et al., 2010). Another pathway to explain why SES could influence procrastination is the variability of self-control ability. Existing evidence indicated that the low SES was a risk factor to make children and students posing less self-control and self-regulation ability (Johnson et al., 2011; Ng-Knight and Schoon, 2017).

In addition, the bidirectional interplay of SES and procrastination could be ascribed to the mediated role of impulsivity. Gustavson et al. (2014, 2015) have demonstrate the robust genetic association between procrastination and impulsivity, with high impulsivity in procrastinators (Gustavson et al., 2014, 2015). Further, it is noteworthy that the high impulsivity could be a reliable predictors for low SES (Assari et al., 2018; Walsh et al., 2019). In this vein, it promotes us to infer that the high impulsivity may make participants prone to procrastinate more for causing poor academic or professional achievements, which in turn, bring about low SES. However, other studies suggested that college students ranging from lower to upper SES backgrounds showed no significant difference in their scores in an university program that aimed to measure academic procrastination (Pychyl et al., 2002). Hence, the relationship between SES and procrastination remains ambiguous, and it is worth exploring through meta-analysis methods.

It has been proposed that cultural differences in norms and values exist in the perception of time that may affect individuals’ evaluation of long-term consequences and risk avoidance, which may thus affect procrastination (Brislin and Kim, 2003). A previous study found that the prevalence of procrastination among British citizens is higher than that among American or Australian citizens, demonstrating that procrastination is more common in Westernized, individualistic, English-speaking countries than in other countries (Ferrari et al., 2005a). In addition, it was found that cultural differences between the United States and Russia are related to procrastination caused by the use of the Internet for social interaction (Doty et al., 2020). Likewise, the same conclusion was found in non-Anglo-Saxon population as well. For example, the problematic procrastination was observed more in Mongols than do of Chinese population (Wang, 2014). In contrast, a group of studies exploring adult procrastination in different cultures found that procrastination is common in every situation, and the procrastination patterns of arousal and avoidance show cross-cultural similarities rather than differences (van Eerde, 2003; Ferrari et al., 2007; Klassen et al., 2009). This claim—that is—no significant cultural differences in procrastination was supported in non-Anglo-Saxon populations, such as Bourau, India and Tibet (Kuang, 2012; Song, 2014). In addition, a study surveying college students from Ukrainian and Slovak revealed that there were no statistically significant differences in procrastination between Ukrainian and Slovak students (Košíková et al., 2020). Overall, it is worthy to explore there were cultural differences for procrastination in the current study.

An interesting association for the procrastination is that delaying off courses irrationality would increase likelihood to remain singe in marital relationship, which caused to a reduction in the size of the family (Moore, 2004). Further, the procrastination was found to be a predictor for divorced rates and it would make individuals hard to obtain romantic relationship (Roberts et al., 2007; Steel, 2010b). A straightforward evidence provided by Steel and Ferrari (2013) indicated the negative association between the procrastination and family size. On the other hand, the high fertility rate was found prominently in individuals with less conscientiousness, which is the key predictor for procrastination (Bouchard, 2005). Thus, the current study is also interested in whether the family size could link to procrastination.

Moreover, educational background has long been regarded as a potential indicator of procrastination tendencies (Ferrari et al., 1995, 2009b; Steel and Ferrari, 2013). Liberal arts students and science students are exposed to different teaching structures and content (Jacobsen, 2006; Van der Wende, 2011) and therefore may differ in their level of procrastination. Conscientiousness is a strong predictor of both academic performance and procrastination (Noftle and Robins, 2007; O’Connor and Paunonen, 2007; Steel, 2007). Later research found a negative relationship between procrastination and academic math performance comparable in strength with that of conscientiousness (Lubbers et al., 2010). Thus, we may deduce that science students procrastinate less than liberal arts students. However, another study investigated whether procrastination is related to student majors and found no significant difference in procrastination tendencies among students of different majors (Zarick and Stonebraker, 2009). Thus, what the association between educational background and procrastination is could be examined by using meta-analysis in the current study.

As conflicting results for the association between procrastination and some demographic characteristics were consistently found, the potential factors to impact these results have also sparked much interests in current study (Pychyl et al., 2002; Özer et al., 2009; Steel and Ferrari, 2013). Svartdal and Steel (2017) have pioneered the examination and revision for five mainstreaming scales, and indicated the variability of psychometric quality for different measurements (Svartdal and Steel, 2017). In addition to this field, results based by heterogeneous measuring tools were found in elsewhere (Dawis, 2000; Marsh et al., 2013). In this vein, it lead us to assume that the conflicting results may be moderated by different scales. Further, procrastination type is also noteworthy to be an alternative moderator. On the basis of temporal motivation theory, the main factor to promote one postponing off is the inadequate motivations (Steel, 2007). A robust body of studies provided solid evidence to claim the interaction effect of gender and motivation, which demonstrated that female posed high intrinsic motivation than male in academic activities (e.g., reading, L2 learning, Rusillo and Arias, 2004; Kissau, 2006; Hakan and Münire, 2014). Despite no straightforward evidence, such interaction may bring about the specific sex-differences between academic procrastination and other ones. Lastly, the educational stage stress should be taken into account to explain the heterogeneous results for sex-differences in procrastination. It is well-known to us for the close association between perceived stress and procrastination, with more stresses for stronger procrastination tendency (Stead et al., 2010; Sirois and Tosti, 2012). On the other hand, more stresses were perceived for students in high educational stages than others, and make undergraduates more prone to procrastinate (Pascoe et al., 2020). Given the significant gender-difference (Barnett et al., 1987), we are interested in probing into how the educational stage may influences procrastination and even the interaction of gender and procrastination.

In the current study, we aim to integrate the results of previous studies of the relationship between procrastination and gender, SES, cultural differences, and educational background and to identify factors that influence their relationship. As aforementioned, we hypothesize that the males may procrastinate more than females. In addition, participants with low SES, western cultural contexts, or majoring in arts may be incline to procrastinate than others. Further, we assumed that the association between procrastination and these demographic architectures would be moderated by measurements/types of procrastination and educational stages.

## Materials and Methods

### Participants and Study Design

This study aimed to investigate the sociodemographic characteristics of procrastination by using a meta-analysis that included gender, socioeconomic status, multiculturalism, family size, and educational background. All the types of procrastination would be in the scope of the current study, such as general procrastination, academic procrastination and decision-making procrastination. Taking into account potential moderated variables or hierarchical natures, the sub-meta-analysis and moderation analyses were used to probe whether these effects were moderated by measurements, procrastination types or other factors. Finally, jackknife analysis was used to validate the robustness of the pooled meta-analytic effects. On balance, this study strove to demonstrate the sociodemographic characteristics of procrastination and unveil the factors that may moderate these effects. This study has been approved by Institutional Review Board (IRB) of Faculty of Educational Science, Sichuan Normal University (China).

### Systematic Meta-Analytic Protocol

The protocol for performing the sociodemographic meta-analysis in the current study was preregistered in the Open Science Framework (OSF) repository beforehand^1^. This study fully adhered to the Preferred Reporting Items for Systematic Reviews and Meta-Analyses (PRISMA) guidelines for literature searching and data extraction (see Supplemental Information).

### Data Source and Search Strategy

The databases to be searched to acquire meta-analytic data were Web of Science (WoS), Science Direct (Elsevier), ProQuest, and Google Scholar. In addition to these international databases, Chinese academic databases were screened as well, including the China National Knowledge Infrastructure (CNKI)^2^, Wangfang^3^, Vip Consult Center^4^ and China Biology Medicine disk (CBMdisc^5^). Searches in these databases were limited to peer-reviewed empirical research published from Jan 2000 to Jan 2021. Despite lack of peer review, dissertations for the full-time Chinese doctoral and master’s degrees were also included in the preliminary data pool once they were determined to be eligible by a modified Newcastle-Ottawa quality assessment (see more details below). Studies published in preprint form were excluded from the data pool for meta-analysis.

### Retrieval Procedure

As a strikingly productive tool, Boolean logic expression (BLE) was drawn upon for literature retrieval. To avoid missing the target, no elimination operator was used in the search. The full search expression in searching the international databases was as follows: (procrastination OR procrastinator OR procrastinate) AND (gender OR sex) OR (socioeconomic status) OR (country OR Han) OR (family size OR single child OR double child) OR (education OR educational backgrounds OR STEM). Likewise, such BLE was also adopted to retrieve target studies in the Chinese databases using Mandarin. To cover all the alternatives for, no exclusion criteria were used in the first searching procedure. Furthermore, to strengthen the search accuracy, the elimination operator was used for re-retrieval. Finally, reference tracking to a hub paper that was cited frequently was undertaken to validate the convergence of this retrieval, such as Steel (2007) and Steel and Ferrari (2013). Specifically, the references cited in hub papers would be reviewed manually, one by one, to validate whether there were missing target papers and to scrutinize whether these potential papers were worth checking for eligibility in the current study.

### Inclusion and Exclusion

To screen available studies for quantitative meta-analysis, the inclusion and exclusion criteria are given here. First, the aims of the screened study should be in line with those of the current study. That is, papers seeking to build upon the link between procrastination and sociodemographic features could be included. Second, these studies should provide adequate effect sizes for meta-analyses, including t values, sample sizes, and descriptive sample information. Third, the procrastination should be measured by widely-used or board-certified scales (e.g., general procrastination scale, and pure procrastination scale). Thus, these studies measuring procrastination from self-made questionnaire or self-report interview would be excluded. Last, the minimum sample size was limited to 30 in each included study to ensure statistical power.

### Data Extraction, Data Coding and Statistics

On the basis of PRISMA protocol, the fundamental information and data were extracted by two independent researchers, including the authors, title, t-statistics, moderators, measurements, and sample size. Further, data coding was performed independently by them. Subsequently, they exchanged data extraction and coding records, and further re-did these procedures to examine for inconsistent results. Finally, once these data were checked for no errors, one researcher inputted them into the CMA software, and another one would double-check whether there were typo independently to validate the correctness of pooling effect size.

Comprehensive meta-analysis (CMA, V3.1) was drawn for meta-analytic statistics in the current study. First, the raw t-statistic (i.e., t-value and sample size) for each included study was estimated for the weighted *r* value and corresponding 95% confidence intervals (CI). Furthermore, all the estimated *r* values were pooled into either a fixed-effect model (FEM) or random-effect model (REM), which was determined by the heterogeneity of the data pool. In this vein, I^2^ and Q^2^ tests were used to evaluate the between-study heterogeneity, with < 20% for low variation, 50% for medium variation and 75% for high variation. A FEM should be adopted if low or medium heterogeneity is detected, while an EFM should be more suitable when high heterogeneity is found (Borenstein et al., 2011). Given the adverse effects of publication bias, Egger’s test and fail-safe N tests were performed to estimate the effects of potential publication bias, with statistical significance in Egger’s test at *p* < 0.05 and *N* > 5k + 10 for predominant publication bias (Fragkos et al., 2014). In addition, the jackknife test was adopted to validate the robustness of the meta-analytic results (Radua and Mataix-Cols, 2009; Frodl and Skokauskas, 2012). This process adopted a leave-one-paper-out (LOPO) scheme to iterate the meta-analysis and further examine whether the targeted significant effects could be maintained.

### Jackknife Analysis and Newcastle-Ottawa Quality Assessment

The Jackknife analysis is to examine the robustness of meta-analytic results by using iteration procedure. In detailed, the meta-analysis would be redone by removing one study included in the datapool, subsequently. This procedure would be iterated until each one was removed once. If the significance of results for all the iteration kept consistent with this meta-analysis, these results would be considered robust enough. Meanwhile, the Newcastle-Ottawa quality assessment was developed to examine the quality of included studies, including five items: balanced gender ratio, sample size, validity, and quality of scales. More details can be found elsewhere (Wells et al., 2014).

## Results

All the coding data and unfolded documents (results) have been uploaded to the OSF repository (see text footnote 1) to promote reproducibility and transparency.

### Included Study and Fundamental Information

**FIGURE 1:**
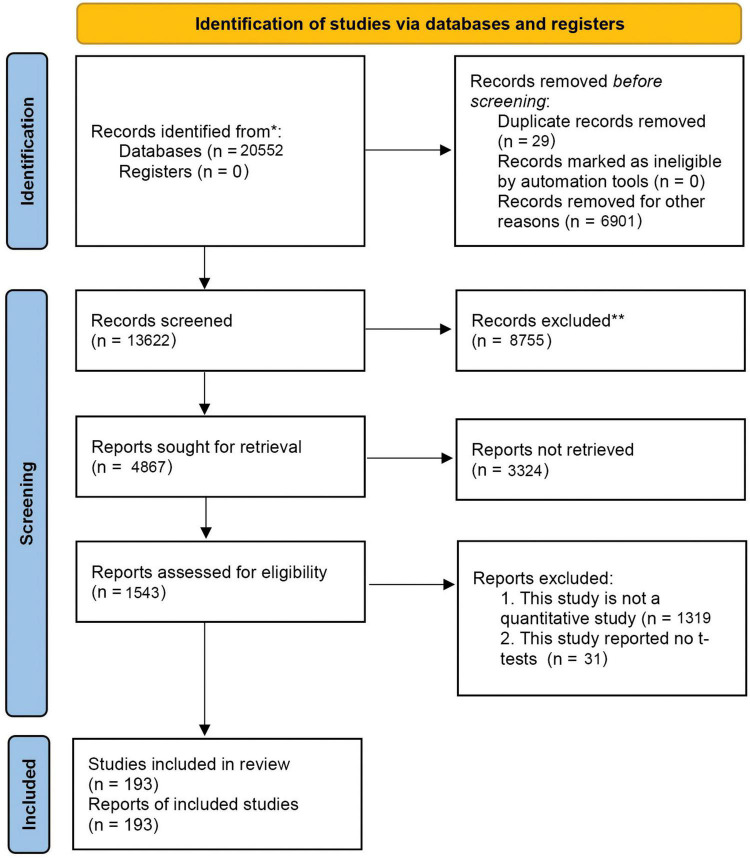
PRISMA 2020 flow diagram for the current meta-analysis.

On the basis of PRISMA protocol, we attempted to search studies on gender-differences in procrastination, and a total of 20,552 studies were retrieved. Afterward, 13,622 studies were retained after duplication checks. Furthermore, 8,755 studies were removed as they fell outside our research aims (e.g., literature review, opinion article) when screening abstracts. Full-text examination was conducted to determine the eligibility of these 3,324 studies. Last, 193 studies were ruled out for formal quantitative meta-analysis because of either a lack of statistical information or non-standardized measurements. In summary, this final meta-analytic model included 193 papers concerning general and academic procrastination (*n* = 102,484) (for more details, see Figure 1). Notwithstanding that, it is unexpected that the vast majority of included studies is derived from mainland China (*k* = 13 and *n* = 3,146 for other countries, *k* = 180 and *n* = 97,604 for Chinese population). Thus, the sample bias should be mentioned for the following analyses. Other meta-analytic models for socioeconomic status, country, family size, and educational background can be found in the SI.

### Synthesized Main Findings

#### Males Procrastinate More Than Females

**FIGURE 2:**
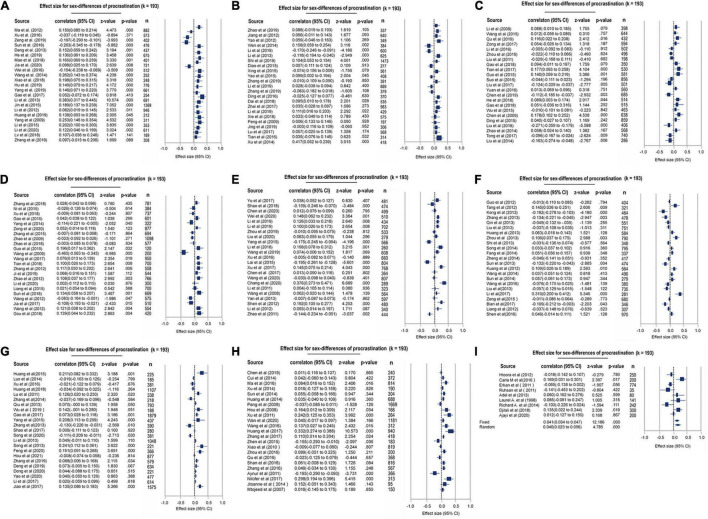
Forest plot for the meta-analytic results toward gender differences of procrastination.

A total of 193 papers (*k* = 193) were included to pool effects for revealing gender differences in both general and academic procrastination, with 102,484 participants [47,901 males (46.73%), 54,583 females (53.27%)]. The findings derived from both Q and I^2^ tests showed a high level of between-study heterogeneity [Q (192) = 1,266.78, *p* < 0.001, I^2^ = 84.84] and thus indicated that the REM is more suitable here. The REM results demonstrated that the procrastination tendency was significantly higher in males than in females (*r* = 0.042, 95% CI: 0.023–0.056, *z* = 4.785, *p* < 0.001) (see Supplementary Table 1 and Figure 2). No publication bias was found in this meta-analysis (Egger’s test *t* = 0.38, *p* = 0.70; Begg test tau = 0.02, *p* = 0.65; fail-safe *N* = 7,226) (see the funnel plot in Supplementary Figure 1). The results of the modified Newcastle-Ottawa quality control assessment demonstrated good quality for the included studies and showed high scorer reliability based on the Spearman test (Qs = 4.48, *r* = 0.99, *p* < 0.001) (see Supplementary Table 1).

#### No Significant Differences in These Procrastination Tendencies Based on Socioeconomic Status

**FIGURE 3:**
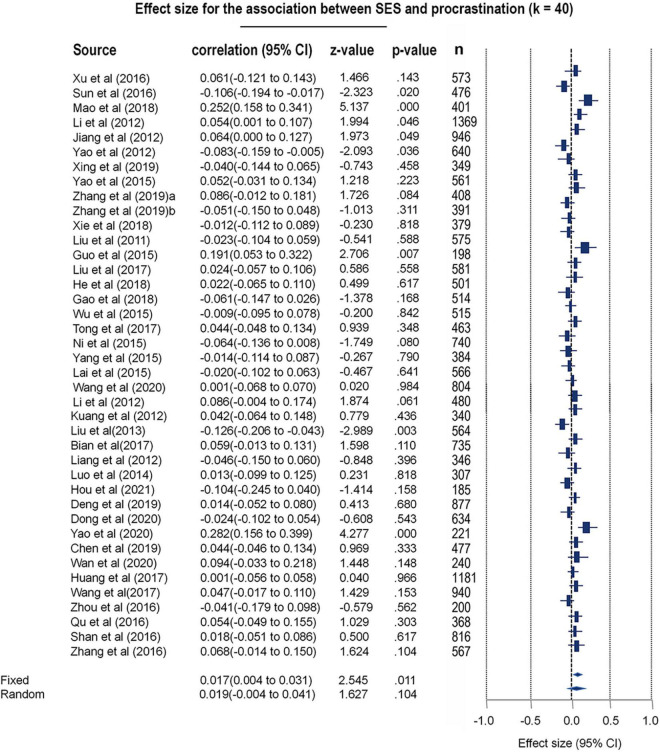
Forest plot for the meta-analytic results toward socioeconomic status differences of procrastination.

Forty papers (*k* = 40) were pooled into a meta-analytic model to probe whether there were differences in procrastination tendencies based on socioeconomic status [*N* = 21,478; 9,540 males (44.41%), 11,938 (55.59%)]. As the between-study heterogeneity was quite high, the REM was adopted for this meta-analysis [Q (39) = 106.74, *p* < 0.001, I^2^ = 63.43]. The results revealed no significant effects of socioeconomic status on procrastination tendencies (*r* = 0.019, 95% CI: −0.004–0.041, *z* = 1.627, *p* = 0.104) (see Figure 3 and Supplementary Table 2). No significant publication bias was found in this meta-analytic model according to Egger’s test and fail-safe N test (Egger’s test *t* = 0.56, *p* = 0.57; Begg test tau = 0.005, *p* = 0.962; fail-safe *N* = 36) (see the funnel plot in Supplementary Figure 2). The included studies were observed to be of good quality on the basis of a modified Newcastle-Ottawa quality control assessment (Qs = 4.53, *r* = 1.00, *p* < 0.001) (see Supplementary Table 3).

#### No Significant Multicultural Differences in These Procrastination Tendencies

**FIGURE 4:**
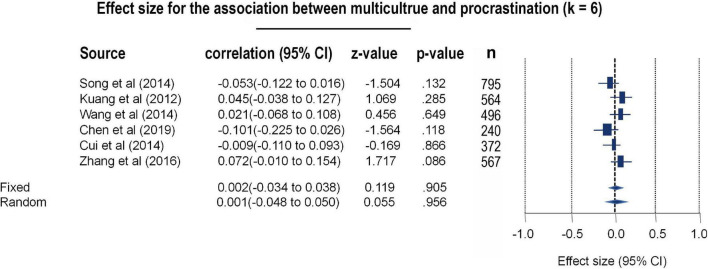
Forest plot for the meta-analytic results toward multi-cultural differences of procrastination.

It should be noted that only Chinese populations were included for this meta-analysis aiming at the cultural differences in procrastination. As the majority and minorities of China posed independent cultures, participants in different ethnic group were considered to undergo different cultural contexts in the current study. Thus, these results derived from this analysis should be considered exploratory and primary. By comparing procrastination between majority and minorities, the six papers (*k* = 6) were included in the meta-analysis to clarify whether procrastination tendencies would vary with respect to multicultural contexts [*N* = 3,091, 1,047 males (33.87%), 2,044 females (66.12%)]. No significant between-study heterogeneity was found in this meta-analysis [Q (5) = 9.02, *p* = 0.108, I^2^ = 44.57]. Instead of the REM, the FEM was deployed to estimate the total effects, and a null finding was revealed (*r* = 0.002, 95% CI: −0.048–0.050, *z* = 0.055, *p* = 0.956) (see Figure 4 and Supplementary Table 4). No significant publication bias was found in this model, either in Egger’s test or in the fail-safe N test (Egger’s test *t* = 0.44, *p* = 0.68; Begg test tau = −0.33, *p* = 0.452; fail-safe *N* = 0) (see the funnel plot in Supplementary Figure 3). Likewise, all the included studies were assessed as eligible (Qs = 3.71, *r* = 0.80, *p* < 0.05) (see Supplementary Table 5).

#### No Evidence to Support the Impact of Family Size on These Procrastination Tendencies

**FIGURE 5:**
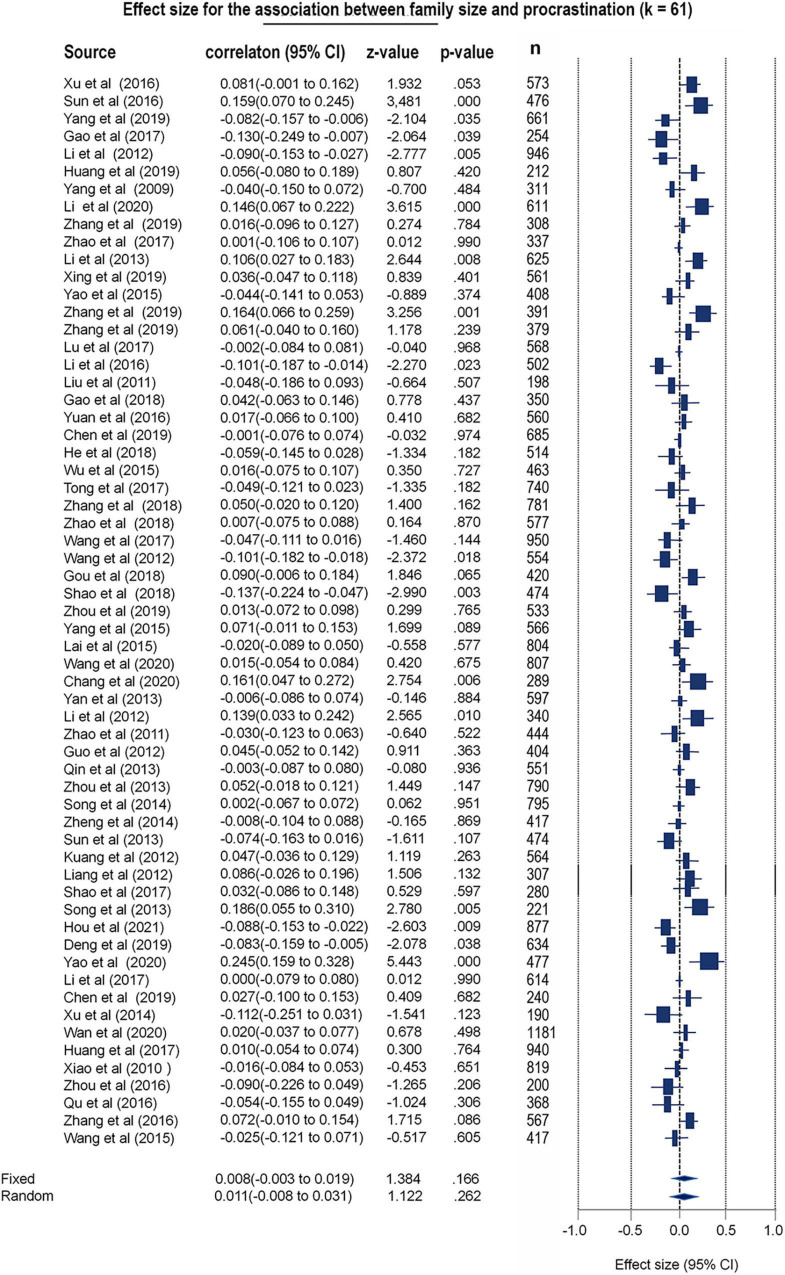
Forest plot for the meta-analytic results toward family size differences of procrastination.

To gain further insights regarding the roles of family size in procrastination tendencies, 61 studies were included in the meta-analytic model. Both Q and I^2^ tests were performed to detect potential between-study heterogeneity, and the results indicated that the REM is more suitable [Q (60) = 182.25, *p* < 0.001, I^2^ = 67.08]. Thus, the REM was used but yielded a null finding (*k* = 61, *r* = 0.011, 95% CI: −0.008–0.031, *z* = 1.122, *p* = 0.262) (see Figure 5 and Supplementary Table 6). Additionally, there was no significant publication bias in this meta-analytic model (Egger’s test *t* = 1.19, *p* = 0.23; Begg test tau = 0.08, *p* = 0.347; fail-safe *N* = 0) (see the funnel plot in Supplementary Figure 4). All the included studies were well validated in terms of quality (Qs = 4.52, *r* = 1.00, *p* < 0.001) (see Supplementary Table 7).

#### No Significant Differences in These Procrastination Tendencies Based on Educational Background

**FIGURE 6:**
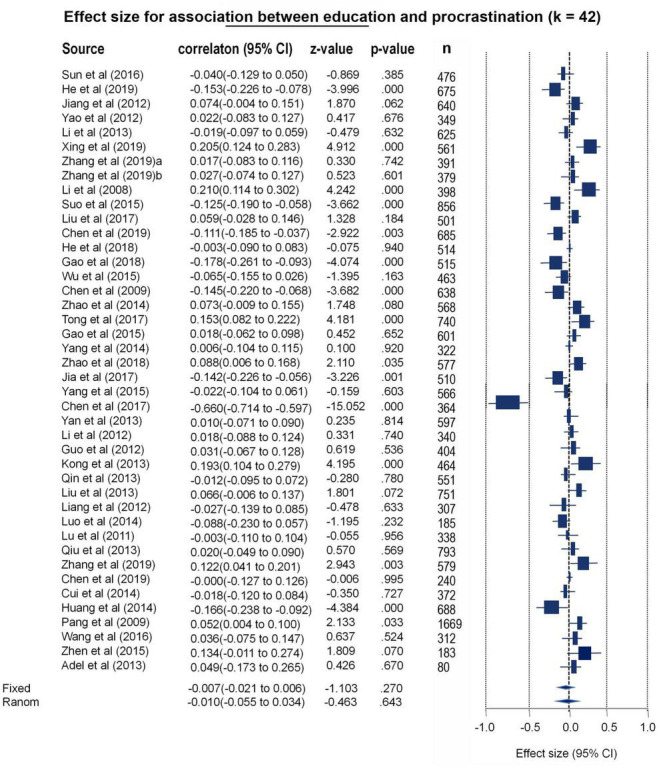
Forest plot for the meta-analytic results toward educational background differences of procrastination.

In the current study, educational background was divided into two types: literature/arts and science. To reveal whether this sociodemographic feature could lead to differences in procrastination tendencies, 42 papers (*k* = 42) were used for a meta-analysis. As between-study heterogeneity was high, the REM was thus used for pooling total effects [Q (41) = 440.05, *p* < 0.001, I^2^ = 90.68]. The results showed a null effect for this association (*r* = −0.010, 95% CI: −0.055–0.034, *z* = −0.46, *p* = 0.643) (see Figure 6 and Supplementary Table 8). As examined by Egger’s and fail-safe N tests, no significant publication bias existed in this model (Egger’s test *t* = 0.37, *p* = 0.70; Begg test tau = −0.03, p = 0.76 fail-safe *N* = 0) (see the funnel plot in Supplementary Figure 5). The included studies were well validated in terms of quality (Qs = 4.62, *r* = 0.99, *p* < 0.001) (see Supplementary Table 9).

### Synthesized Results of Moderated and Sub-Group Analysis

#### Gender Difference Was Moderated by Various Measurements

High between-study heterogeneity has long been acknowledged to be implicated to potential moderators. In this vein, the current study conducted moderated analysis by using mixed effects analysis. Results indicated that the gender difference of procrastination tendency was moderated by various measurements significantly (point estimate = 0.042, 95% CI: 0.027–0.057, *z* = 5.474, *p* < 0.001). Sub-group analysis was conducted to clarify this moderated effects, and showed the high gender differences effects in Aitken Procrastination Inventory (API, point estimate = 0.035, 95% CI: −0.011–0.081), The Procrastination Assessment Scale–Students (PASS, point estimate = 0.056, 95% CI: 0.029–0.083), and the Academic Procrastination Questionnaire—Middle School Student (APQ-MSS, point estimate = 0.072, 95% CI: 0.031–0.112), as well appeared low effects in General Procrastination Scale (GPS, point estimate = 0.018, 95% CI: −0.019–0.056) and Tuckman Procrastination Scale (TPS, point estimate = 0.026, 95% CI: −0.027–0.078).

#### Gender Differences Had a Stronger Effect on Academic Procrastination Than on General Procrastination

Further moderated analysis revealed the moderating role of procrastination types, including academic procrastination and general procrastination (point estimate = 0.042, 95% CI: 0.026–0.058, *z* = 5.139, *p* < 0.001). Subgroup analysis demonstrated prominently larger effects on academic procrastination (point estimate = 0.047, 95% CI: 0.030–0.065) than on general procrastination (point estimate = 0.019, 95% CI: −0.019 to −0.057).

#### Graduate Students Showed Stronger Gender Differences Related to These Procrastination Tendencies

As large effects on academic procrastination were found, we performed a sub-sub-meta-analysis to probe whether such effects would be moderated by the different stages of education, including primary school, junior school, high school, undergraduate and postgraduate levels. The results illustrated the significant moderating effects of educational stages on gender differences related to procrastination tendencies (point estimate = 0.041, 95% CI: 0.026–0.056, *z* = 5.300, *p* < 0.001). Further *post hoc* analysis demonstrated larger effects for undergraduate students (point estimate = 0.040, 95% CI: 0.018–0.062) and graduate students (point estimate = 0.138, 95% CI: 0.027–0.246) than students of other education levels (see Table 1).

**TABLE 1:** Summary of moderated effects of identity for the association between gender and procrastination.

Groups	Number Studies	Effect size and 95%interval			Test of null (2-tail)		Heterogeneity		
		Point estimate	Lower limit	Upper limit	Z-value	*P*-value	Q-value	df(Q)	*P*-value
Adult	3	0.039	−0.043	0.121	0.931	0.352			
College student	95	0.040	0.018	0.062	3.592	0.000			
Graduate	9	0.138	0.027	0.246	2.427	0.015			
High school	18	0.080	0.042	0.117	4.167	0.000			
Junior school	37	0.022	−0.012	0.056	1.280	0.200			
Primary school	31	0.004	−0.044	0.052	0.165	0.869			
Total between							10.599	5	0.060
Overall	193	0.041	0.026	0.056	5.300	0.000			

### Results of Jackknife Examination of Robustness

All the statistical results were validated to pass the jackknife test for examining robustness, showing no outliers (see Supplementary Table 10).

## Discussion

The current study performed sociodemographic meta-analyses to synthesize the results of previous studies on the relationship between general/academic procrastination and gender, SES, cultural differences, and educational background, and to explore potential factors that affect this relationship. By including 193 quantitative studies with 102,484 participants, the results showed that the males procrastinate more than females with moderate effect size (*r* = 0.042, 95% CI: 0.023–0.056, *z* = 4.785, *p* < 0.001) for general and academic procrastination. In addition, by using a small sample size (*n* = 21,478, *k* = 40 for SES; *n* = 3,091, *k* = 6 for multi-cultural contexts; *n* = 32,096, *k* = 61 for family size; *n* = 21,767, *k* = 42 for educational stage), no significant effects were found to support the sociodemographic association between procrastination and SES, multi-cultural contexts, family size, educational stage (*r* = 0.02, *p* = 0.10 for SES; *r* = 0.002, *p* = 0.97 for multi-cultural contexts; *r* = 0.01, *p* = 0.26 for family size; *r* = −0.01, *p* = 0.64 for educational stage). Further, we found that various measurements of procrastination, procrastination types, and educational stages significantly moderated this relationship (*Q* = 0.04, *p* < 0.001 for different measurements; *Q* = 0.04, *p* < 0.001 for procrastination types; *Q* = 0.04, *p* < 0.001 for educational stages). *Post hoc* analysis demonstrated the high effect size for gender-differences of procrastination in API (*Q* = 0.035, *p* < 0.001) and PASS (*Q* = 0.056, *p* < 0.001) and revealed the low effect size by using GPS (*Q* = 0.018, *p* > 0.05) and TPS (*Q* = 0.026, *p* > 0.05). Also, the effect size for gender-differences of procrastination was found in academic procrastination (*Q* = 0.042, *p* < 0.001) compared to general procrastination (*Q* = 0.019, *p* > 0.05). Lastly, graduate students were found higher effect size than others significantly (*Q* = 0.138, *p* < 0.001). Thus, this study could lead us to draw a conclusion that males procrastinate more than females in both general and academic procrastination, especially in Chinese contexts. Further, this relationship may be moderated by the measurements, type of procrastination and academic status in the almost Chinese samples. Further, this relationship may be moderated by the measurements, type of procrastination and academic status. On the other hand, by using small-size samples, there were no enough evidence to claim the sociodemographic association of procrastination for SES, multi-cultural contexts, family size and educational stages.

### Gender Is Significantly Correlated With General and Academic Procrastination

In particular, this study provided robust statistical evidence that males procrastinate more than females (*n* = 102,484, *k* = 193; *r* = 0.042, 95%, *p* < 0.001) in both general and academic procrastination, which was consistent with previous investigations suggesting a relationship between them (Pychyl et al., 2000; Steel, 2007; Gröpel and Steel, 2008). There might be promising evidence suggesting a causal role of demographic features (i.e., gender) in procrastination. Males were found to possess a lower level of self-control, which is a key determinant of procrastination (Tewksbury and Higgins, 2006; Ward et al., 2018). As a result, males may tend to procrastinate more due to a lack of goal-directed processing ability and an inability to suppress tempting stimuli (Pychyl et al., 2000; Ferrari, 2001; Steel, 2007; Steel and Klingsieck, 2016). Similarly, males also have a higher level of impulsivity than do females (Cross et al., 2011). A large number of studies have demonstrated that procrastination is positively associated with impulsivity from the behavior, neural variance and behavioral genetics perspectives (Steel and König, 2006; Gustavson et al., 2014; Liu and Feng, 2017), suggesting that males may procrastinate more than females as a result of intrinsic neurobiological factors. Furthermore, a previous meta-analysis found that females score higher on effortful control than males and that effortful control is closely related to procrastination as well (Else-Quest et al., 2006; Lian et al., 2018). This may explain why females procrastinate less than males. On the other hand, existing studies have provided insights into the evolutionary origins of procrastination, suggesting that procrastination can be considered a strategy frequently used throughout life to deal with unpredictable circumstances (Steel, 2007; Chen and Chang, 2016; Chen and Qu, 2017). Compared to females, males were identified to be more sensitive to unpredictable environments, in which they are able to adapt and quickly develop a strategy to succeed using their past experience (Del Giudice, 2009; Jonason et al., 2017). Thus, males’ procrastination tendencies may be stronger than those of females as an evolutionary consequence. Notwithstanding that, the external ecological validity should be mentioned to discuss above results. Notably, there were no any statistical considerations aiming at the ecological validity of this meta-analysis though the substantial heterogeneity for these included studies was found (Andrade, 2018). Despite statistical significance, above explanations to support our findings that the males procrastinate more than females were largely grounded *post hoc* evidence. There were evidence not enough to validate whether this conclusion could be generalizable elsewhere. Given that, extending this conclusion should be quite careful. On balance, the results demonstrated that males procrastinate more than females, not only because of their low levels of self-control and effortful control and high levels of impulsivity but also because of human evolutionary influences.

### Null Findings Were Observed for the Association Between Procrastination and SES, Multicultural Differences, Family Size and Educational Background

However, the hypotheses in present study has not been fully confirmed. For instance, no significant correlation was found between SES and procrastination in the limited sample size (*n* = 21,478, *k* = 40, *r* = 0.02, *p* = 0.10). One possible explanation is that SES mediates procrastination through other factors, such as parenting style and self-efficacy (Wäschle et al., 2014). Another explanation may be that the high heterogeneity confounded the meta-analytic effects as unaccountable random factors. In addition, the results showed no significant correlation between multicultural differences and procrastination. As reviewed by Steel (2007), procrastination is a personality-like trait and is relatively stable in cross-cultural contexts. Furthermore, there was no significant association between family size and procrastination. On the one hand, procrastination may manifest in postponing childbirth (Steel, 2010b; Steel and Ferrari, 2013; Schippers et al., 2015), which may complicate the association between procrastination and the number of children. On the other hand, unplanned pregnancies are associated with impulsiveness, which is a strong predictor of procrastination (Kahn et al., 2002). Thus, we posited that the association between family size and procrastination may develop in a more complicated, non-linear manner. In addition, no correlation was found between educational background and procrastination, which was consistent with previous research suggesting that majoring in liberal arts or science is not a determinant of procrastination (Seker, 2015). Collectively, despite the failure to validate all the hypotheses, the current study provides robust meta-analytic evidence to substantiate the association between gender and procrastination.

### Heterogeneous Psychometric Tools, Types and Educational Stages Biased the Conclusion Regarding the Association Between Gender and Procrastination

Existing studies have not well established the connection between gender and procrastination. Some studies demonstrated that gender was significantly correlated with procrastination (Gröpel and Steel, 2008), while others revealed a null correlation between them (Ferrari, 1989; Ferrari et al., 2005b). Thus, it is valuable to explain why there were different results for the association between procrastination and gender. To this end, the current study performed moderation analysis to clarify the potential factors resulting in inconsistent results. The results of the moderation analysis showed that the heterogeneity in the measurement tools, procrastination types, and educational stages could moderate the association between gender and procrastination.

First, the current study noted the different sensitivity levels of tools for measuring procrastination, with strong effects for the association between gender and procrastination in terms of API, PASS and APQ scores and weaker effects on GPS and TPS scores. Thus, to obtain reliable results, widely used, revised and well-established scales or questionnaires, such as the GPS and PPS (i.e., pure procrastination scale), should be adopted. In addition, the psychometric properties of different scales should be validated robustly before attempting to measure procrastination. Despite the solid theoretical basis, there was weak evidence to support the psychometric robustness of some of the scales.

Another important finding derived from this sub-meta-analysis was that the observed association between procrastination and gender was influenced by procrastination type, with the largest effects on academic procrastination. It has been suggested that females show a fear of strangers and unfamiliar events at an earlier age than males (Archer, 1991). Additionally, female students tend not to procrastinate in their academic tasks because of a fear of achieving low course grades (Özer et al., 2009). On the other hand, male students more frequently reported that they procrastinated in their studies due to risk taking and resisting control (Lippa, 2002). In addition, male students are more impulsive than adults, suggesting that they may be more inclined to delay academic tasks (Steinberg et al., 2008; Duckworth et al., 2013). One more alternative explanation worthy to note that the procrastination type moderated the gender-difference of procrastination by age. Beutel et al. (2016) provided solid evidence to demonstrate that the gender-differences of procrastination were found only in the young population (ages from 14 to 29) instead of the overall large-scale sample (Beutel et al., 2016). It may indicate that the academic procrastination was frequently found in young students and it thus let the gender-differences of procrastination more obvious than others. Overall, these findings may indirectly indicate that gender differences more strongly affect general procrastination than academic procrastination.

Last, the sub-meta-analysis further revealed that the observed relationship between procrastination and gender is influenced by educational stage, and the influence of undergraduate students is stronger than that of students at other levels. Previous studies have suggested that undergraduate/graduate students procrastinate more than high school or primary school students, because undergraduate/graduate students have more freedom in terms of time and content to complete academic tasks than high school students do (Milgram et al., 1993; Özer and Saçkes, 2011). Thus, compared to students at other educational stages, undergraduate students were likely to be influenced by more distractors and devalued the utility of rewards for completing academic tasks, which made those students more inclined to procrastinate. Additionally, taking into account neuroendocrine factors, females are more fearful and avoidant than males as part of hormonal factors in the late teens (Coates and Wolfe, 1995). In this vein, as procrastination has been proven to be correlated with avoidant motivation (e.g., Steel, 2007, 2010a), the moderating role of educational stage on the association between gender and procrastination could be explained by the neuroendocrine variation between males and females.

### Limitation and Future Directions

Although this study here revealed demographic characteristics of procrastination, several limitations should warrant cautions. It is worthy to note that a portion of meta-analyses just included the limited number of existing studies, such as the cross-cultural differences of procrastination. Thus, given the marginal sample size, these conclusions should be preliminary and exploratory, as well should be interpreted more cautiously. Another one should bear in mind that the included studies mainly focus on Chinese population (93%). Given the significant sample biases, it should warrant some cautions when these conclusions would be extended elsewhere. Also, it is merit to test the generalizability of the current study in more diverse samples. It is worthy to note that a portion of meta-analyses just included the limited number of existing studies, such as the cross-cultural differences of procrastination. Further, the robustness of results that derived from cross-cultural differences was challenged by sample representation as well. Taken both reasons together, the conclusions for this analysis should be preliminary and exploratory, as well should be interpreted more cautiously. In addition, we found considerable heterogeneity in these meta-analyses. Although we discovered some moderating factors, more factors are worth studying to account for this heterogeneity. Furthermore, the sample representation was somewhat inadequate (e.g., much more Chinese participants than others), which suggests the need for future research using a representative sample to achieve unbiased results. Lastly, as limited by existing literature, it lacks a portion of types of procrastination in meta-analytic model, such as bedtime procrastination and health procrastination. Thus, it is merit to include multiple procrastination types for providing more robust evidence in future study.

## Conclusion

In summary, this meta-analysis suggested that males procrastinate more than females. In addition, the current study revealed no significant association between procrastination and other sociodemographic features, including socioeconomic status, cultural differences, family size and educational background. Overall, the main strength of our meta-analysis study yields insights into the sociodemographic characteristics of procrastination and the factors that may moderate these effects. Given the association between procrastination and societal ailments (e.g., delayed medical treatment), identifying sociodemographic characteristics associated with procrastination would be valuable for directing public policies aimed at prevention.

## Data Availability Statement

All the raw data, scripts, figures and materials can be accessible at OSF repository (https://osf.io/c928r/).

## Ethics Statement

The studies involving human participants were reviewed and approved by IRB of Faculty of Educational Science, Sichuan Normal University (China). The patients/participants provided their written informed consent to participate in this study.

## Author Contributions

DL: conceptualization, methodology, software, writing—original draft and visualization, supervision, project administration, and funding acquisition. YH and YT: writing—review and editing, methodology, and validation. All authors contributed to the article and approved the submitted version.

## Conflict of Interest

The authors declare that the research was conducted in the absence of any commercial or financial relationships that could be construed as a potential conflict of interest.

## Publisher’s Note

All claims expressed in this article are solely those of the authors and do not necessarily represent those of their affiliated organizations, or those of the publisher, the editors and the reviewers. Any product that may be evaluated in this article, or claim that may be made by its manufacturer, is not guaranteed or endorsed by the publisher.

## Footnotes

1
https://osf.io/c928r/

2
http://www.cnki.net

3
http://www.wanfangdata.com.cn

4
http://www.cqvip.com/

5
http://www.sinomed.ac.cn/
