# Supplementary material for: Gender, Socioeconomic Status, Cultural Differences, Education, Family Size and Procrastination: A Sociodemographic Meta-Analysis
Source: Front Psychol. 2022 Jan 5;12:719425. doi: 10.3389/fpsyg.2021.719425 (PMC8766341; doi:10.3389/fpsyg.2021.719425)
Supplement: Supplementary file 1 [file Table_1.doc]

| Author (year) | Gender, male (female) | Country/Areas | Age (Mean ± S.D) | Assessments | Identity | Type | Quality scores |
| --- | --- | --- | --- | --- | --- | --- | --- |
| Adel et al (2013) | 40(40) | Egypt | 19.1 ± 9.5 | Aitken | college student | Academic Procrastination | 4 |
| Ajayi et al (2020) | 100(100) | Nigeria | _ | Tuckman | college student | Academic Procrastination | 3.5 |
| Aynur et al (2011) | 114(251) | Turkey | _ | Aitken | college student | Academic Procrastination | 4 |
| Bian et al(2017) | 154(192) | China | _ | Lay | college student | General Procrastination | 5 |
| Carla M et al(2016） | 85(115) | Philippines | _ | Tuckman | college student | Academic Procrastination | 3 |
| Chang et al (2020) | 119(203) | China | - | Lay | primary school student | General Procrastination | 5 |
| Chen et al (2017) | 182(182) | China | - | PASS | college student | Academic Procrastination | 5 |
| Chen et al (2009) | 277(361) | China | - | PASS | college student | Academic Procrastination | 5 |
| Chen et al (2019) | 294(391) | China | 19.4 | PASS | College student | Academic Procrastination | 5 |
| Chen et al (2019) | 108(132) | China | _ | PASS | college student | Academic Procrastination | 4 |
| Chen et al (2020) | 237(262) | China | - | Aitken | junior school student | Academic Procrastination | 5 |
| Cui et al (2014) | 29(143) | China | _ | PASS | college student | Academic Procrastination | 4 |
| Dai et al (2018) | 263(272) | China | - | Lay | Primary student | General Procrastination | 5 |
| Deng et al (2013) | 360(383) | China | - | Aitken | Middle school student | Academic Procrastination | 5 |
| Deng et al (2019) | 153(481) | China | 20 ± 1 | PASS | college student | Academic Procrastination | 3 |
| Diao et al (2015) | 73(204) | China | - | PASS | Graduate student | Academic Procrastination | 3 |
| Ding et al (2015) | 406(453) | China | - | Aitken | college student | Academic Procrastination | 5 |
| Ding et al (2016) | 157(213) | China | - | Liao Yanran | Adult | Academic Procrastination | 5 |
| Djilali et al (2018) | 150(150) | Arab | 19.51 ± 1.77 | Aitken | college student | Academic Procrastination | 5 |
| Dong et al (2020) | 57(164) | China | _ | PASS | college student | Academic Procrastination | 3 |
| Erkan et al（2011） | 276(498) | Turkey | 21.43 ± 2.15 | Aitken | college student | Academic Procrastination | 4 |
| Fang et al (2014) | 187(150) | China | - | PASS | college student | Academic Procrastination | 5 |
| Feng et al (2020) | 178(188) | China | _ | Lay | primary school student | General Procrastination | 5 |
| Gao et al (2015) | 219(322) | China | - | PASS | college student | Academic Procrastination | 5 |
| Gao et al (2017) | 150(104) | China | - | Lay | Primary student | General Procrastination | 4 |
| Gao et al (2017) | 992(887) | China | _ | Zuo Yanmei | high school student | Academic Procrastination | 5 |
| Gao et al (2018) | 174(176) | China | - | Lay | College student | General Procrastination | 5 |
| Gao et al (2018) | 268(241) | China | - | PASS | College student | Academic Procrastination | 5 |
| Gou et al (2018) | 235(185) | China | - | Lay | primary school student | General Procrastination | 5 |
| Guo et al (2012) | 280(124) | China | - | Lay | graduate student | General Procrastination | 4 |
| Guo et al (2015) | 307(274) | China | - | PASS | Junior school student | Academic Procrastination | 5 |
| Guo et al (2015) | 51(69) | China | - | Lay | college student | General Procrastination | 3 |
| He et al (2018) | 160(354) | China | - | Aitken | College student | Academic Procrastination | 4 |
| He et al (2019) | 271(332) | China | 15.32 ± 2.51 | Aitken | Middle school student | Academic Procrastination | 5 |
| Hoora et al (2012) | 129(121) | Iran | _ | Aitken | high school student | Academic Procrastination | 3.5 |
| Hou et al (2008) | 50(116) | China | _ | PASS | college student | Academic Procrastination | 3 |
| Hou et al (2021) | 377(500) | China | _ | PASS | college student | Academic Procrastination | 5 |
| Hu et al (2019) | 229(211) | China | - | Lay | Primary student | General Procrastination | 5 |
| Huang et al (2013) | 282(302) | China | - | Milgram | primary school student | Academic Procrastination | 5 |
| Huang et al (2014) | 200(488) | China | _ | Tuckman | college student | Academic Procrastination | 5 |
| Huang et al (2017) | 391(549) | China | 20.54 ± 1.41 | Lay | college student | General Procrastination | 4 |
| Huang et al (2019) | 116(96) | China | - | Milgram | Primary student | Academic Procrastination | 4 |
| Huang et al(2015) | 105(120) | China | _ | Milgram | college student | Academic Procrastination | 2 |
| Huang et al(2018) | 222(885) | China | 20.53 ± 1.53 | PASS | college student | Academic Procrastination | 4 |
| Ji et al (2019) | 307(237) | China | - | Lay | junior school student | General Procrastination | 5 |
| Jia et al (2017) | 185(325) | China | - | PASS | graduate student | Academic Procrastination | 5 |
| Jia et al (2020) | 302(419) | China | 20.01 ± 1.56 | PASS | College student | Academic Procrastination | 5 |
| Jiang et al (2012) | 212(403) | China | - | Lay | College student | General Procrastination | 4 |
| Jiao et al (2017) | 259(1316) | China | _ | PASS | college student | Academic Procrastination | 3 |
| Jin et al (2015) | 687(660) | China | - | Milgram | Primary student | Academic Procrastination | 4 |
| Jing et al (2019) | 148(158) | China | 14.14 ± 10.23 | Liao Yanran | Adult | Academic Procrastination | 5 |
| Joanne et al（2014） | 26(66) | Germany | _ | Aitken | college student | Academic Procrastination | 2.5 |
| Kong et al (2013) | 201(263) | China | - | PASS | college student | Academic Procrastination | 5 |
| Kuang et al (2012) | 330(598) | China | - | Lay | college student | General Procrastination | 5 |
| Lai et al (2015) | 466(338) | China | - | PASS | college student | Academic Procrastination | 5 |
| Laurel A. et al (1998) | 54(87) | America | 24.5 ± 7.4 | Lay | college student | General Procrastination | 4 |
| Li et al (2008) | 183(215) | China | - | PASS | High school student | Academic Procrastination | 5 |
| Li et al (2011) | 141(182) | China | - | PASS | junior school student | Academic Procrastination | 5 |
| Li et al (2012) | 479(466) | China | - | Milgram | Primary student | Academic Procrastination | 5 |
| Li et al (2012) | 113(227) | China | - | Aitken | college student | Academic Procrastination | 4 |
| Li et al (2013) | 350(308) | China | - | Lay | Primary student | General Procrastination | 5 |
| Li et al (2013) | 250(375) | China | - | PASS | Graduate student | Academic Procrastination | 5 |
| Li et al (2013) | 532(516) | China | _ | Aitken | junior school student | Academic Procrastination | 5 |
| Li et al (2015) | 185(168) | China | - | Lay | Primary student | General Procrastination | 5 |
| Li et al (2016) | 193(309) | China | - | Aitken | High school student | Academic Procrastination | 5 |
| Li et al (2016) | 134(126) | China | - | Lay | primary school student | General Procrastination | 4 |
| Li et al (2017) | 194(420) | China | _ | PASS | college student | Academic Procrastination | 4 |
| Li et al (2019) | 346(254) | China | - | Tuckman | Graduate student | Academic Procrastination | 5 |
| Li et al (2019) | 404(484) | China | - | PASS | High school student | Academic Procrastination | 5 |
| Li et al (2019) | 191(238) | China | - | Ran Hong | Middle school student | Academic Procrastination | 5 |
| Li et al (2019) | 209(225) | China | 12.03 ± 0.85 | Lay | primary school student | General Procrastination | 5 |
| Li et al (2019) | 380(322) | China | - | Lay | primary school student | General Procrastination | 4 |
| Li et al (2020) | 330(281) | China | - | Lay | Primary student | General Procrastination | 5 |
| Li et al (2020) | 148(137) | China | - | PASS | junior school student | Academic Procrastination | 4 |
| Li et al(2017) | 159(122) | China | 16 ± 1.117 | PASS | junior school student | Academic Procrastination | 4 |
| Liang et al (2012) | 146(161) | China | _ | Aitken | college student | Academic Procrastination | 5 |
| Liang et al (2015) | 316(384) | China | - | PASS | junior school student | Academic Procrastination | 5 |
| Liu et al (2011) | 67(131) | China | - | Lay | College student | General Procrastination | 3 |
| Liu et al (2013) | 365(386) | China | - | Lay | college student | General Procrastination | 5 |
| Liu et al (2014) | 139(147) | China | - | PASS | college student | Academic Procrastination | 5 |
| Liu et al (2017) | 225(276) | China | - | PASS | College student | Academic Procrastination | 5 |
| Liu et al (2018) | 136(270) | China | - | PASS | college student | Academic Procrastination | 4 |
| Liu et al (2020) | 139(140) | China | - | Lay | primary school student | General Procrastination | 4 |
| Liu et al(2013) | 421(314) | China | _ | Aitken | junior school student | Academic Procrastination | 5 |
| Lu et al (2011) | 163(175) | China | _ | Lay | college student | General Procrastination | 5 |
| Lu et al (2017) | 315(252) | China | 16.2 ± 0.9 | Zuo Yanmei | High school student | Academic Procrastination | 5 |
| Luo et al (2014) | 72(113) | China | _ | PASS | college student | Academic Procrastination | 3 |
| Lv et al (2018) | 106(83) | China | - | Lay | Primary student | General Procrastination | 4 |
| Ma et al (2012) | 399(483) | China | - | Milgram | Junior school student | Academic Procrastination | 5 |
| Ma et al (2016) | 206(200) | China | _ | Lay | primary school student | General Procrastination | 5 |
| Mao et al (2018) | 194(207) | China | - | PASS | College students | Academic Procrastination | 5 |
| Mojeed et al (2007) | 85(65) | Botswana | 24.6 | Tuckman | college student | Academic Procrastination | 4 |
| Ni et al (2015) | 200(184) | China | - | Lay | college student | General Procrastination | 5 |
| Nilüfer et al (2017) | 169(144) | Turkey | 44.75 ± 13.08 | Aitken | college student | Academic Procrastination | 5 |
| Pang et al (2009) | 742(882) | China | 20.26 | PASS | college student | Academic Procrastination | 4 |
| Peng et al (2009) | 116(81) | China | - | Lay | Adult | General Procrastination | 4 |
| Qin et al (2013) | 316(235) | China | - | PASS | college student | Academic Procrastination | 5 |
| Qiu et al (2013) | 385(408) | China | _ | Ran Hong | high school student | Academic Procrastination | 5 |
| Qu et al (2016) | 170(198) | China | _ | Aitken | college student | Academic Procrastination | 5 |
| Qu et al (2019) | 208(224) | China | - | Zuo Yanmei | High school student | Academic Procrastination | 5 |
| Richard A. et al (2014) | 128(114) | Nigeria | 35.64 ± 10.66 | Tuckman | college student | Academic Procrastination | 3.5 |
| Ruhsan et al（2011) | 18(17) | America | _ | PASS | college student | Academic Procrastination | 3 |
| Shan et al (2016) | 436(380) | China | 20 ± 2 | Lay | college student | General Procrastination | 5 |
| Shao et al (2017) | 133(147) | China | _ | Aitken | college student | Academic Procrastination | 4 |
| Shao et al (2018) | 203(271) | China | - | Aitken | high school student | Academic Procrastination | 5 |
| Shen et al (2012) | 248(220) | China | - | PASS | junior school student | Academic Procrastination | 5 |
| Shen et al(2016) | 219(751) | China | _ | Lay | college student | General Procrastination | 4 |
| Shi et al (2013) | 156(192) | China | - | Ran Hong | high school student | Academic Procrastination | 5 |
| Shi et al (2019) | 805(668) | China | - | Lay | Primary student | General Procrastination | 5 |
| Song et al (2013) | 96(125) | China | _ | PASS | junior school student | Academic Procrastination | 3 |
| Song et al (2014) | 200(488) | China | - | PASS | college student | Academic Procrastination | 4 |
| Song et al (2020) | 147(234) | China | _ | Lay | college student | General Procrastination | 5 |
| Sun et al (2013) | 230(244) | China | - | PASS | junior school student | Academic Procrastination | 4 |
| Sun et al (2014) | 108 (189) | China | 20.4 ± 2.3 | Tuckman | college student | Academic Procrastination | 4 |
| Sun et al (2014) | 108(189) | China | 20.4 ± 2.3 | Tuckman | college student | Academic Procrastination | 4 |
| Sun et al (2016) | 184(292) | China | - | Zuo Yanmei | High school student | Academic Procrastination | 5 |
| Sun et al (2018) | 351(318) | China | - | Zuo Yanmei | junior school student | Academic Procrastination | 5 |
| Suo et al (2015) | 337(519) | China | - | PASS | College student | Academic Procrastination | 4 |
| Tang et al (2012) | 187(131) | China | - | PASS | junior school student | Academic Procrastination | 5 |
| Tian et al (2016) | 149(165) | China | - | Ran Hong | High school student | Academic Procrastination | 5 |
| Tian et al (2017) | 255(275) | China | - | Aitken | Junior school student | Academic Procrastination | 5 |
| Tian et al (2018) | 399(301) | China | - | Zuo Yanmei | junior school student | Academic Procrastination | 5 |
| Tong et al (2017) | 377(363) | China | - | PASS | college student | Academic Procrastination | 5 |
| Wan et al (2020) | 611(570) | China | _ | Lay | primary school student | General Procrastination | 5 |
| Wang et al (2008) | 300(264) | China | - | Tuckman | junior school student | Academic Procrastination | 5 |
| Wang et al (2009) | 102(98) | China | - | PASS | junior school student | Academic Procrastination | 4 |
| Wang et al (2012) | 313(62) | China | - | Zuo Yanmei | junior school student | Academic Procrastination | 5 |
| Wang et al (2012) | 285(269) | China | - | Milgram | primary school student | Academic Procrastination | 4 |
| Wang et al (2014) | 121(131) | China | - | Zuo Yanmei | Junior school student | Academic Procrastination | 4 |
| Wang et al (2014) | 206(290) | China | - | PASS | college student | Academic Procrastination | 5 |
| Wang et al (2015) | 301(343) | China | 16.24 ± 1.16 | Aitken | High school student | Academic Procrastination | 5 |
| Wang et al (2016) | 198(182) | China | 20.5 | Aitken | college student | Academic Procrastination | 5 |
| Wang et al (2016) | 169(143) | China | 21 ± 3 | Lay | college student | General Procrastination | 5 |
| Wang et al (2017) | 476(474) | China | - | Ran Hong | junior school student | Academic Procrastination | 5 |
| Wang et al (2019) | 295(313) | China | - | Ran Hong | high school student | Academic Procrastination | 5 |
| Wang et al (2020) | 326(481) | China | - | Aitken | college student | Academic Procrastination | 5 |
| Wei et al (2016） | 160(352) | China | - | PASS | Graduate student | Academic Procrastination | 4 |
| Wei et al (2020) | 280(230) | China | - | Lay | primary school student | General Procrastination | 5 |
| Wen et al (2014) | 181(203) | China | - | Milgram | Graduate student | Academic Procrastination | 5 |
| Wu et al (2015) | 175(288) | China | - | PASS | College student | Academic Procrastination | 5 |
| Wu et al（2019） | 95(93) | China | _ | PASS | primary school student | Academic Procrastination | 3 |
| Xiao et al (2010） | 385(407) | China | 20.94 ± 2.47 | Lay | college student | General Procrastination | 5 |
| Xiao et al (2018) | 129(119) | China | - | Lay | Primary student | General Procrastination | 4 |
| Xie et al (2018) | 385(190) | China | - | Zuo Yanmei | Middle school student | Academic Procrastination | 4 |
| Xing et al (2019) | 282(279) | China | - | PASS | College student | Academic Procrastination | 5 |
| Xu et al (2017) | 379(389) | China | - | PASS | college student | Academic Procrastination | 5 |
| Xu et al (2011) | 140(124) | China | _ | Tuckman | junior school student | Academic Procrastination | 4 |
| Xu et al (2014) | 191(227) | China | - | Zuo Yanmei | High school student | Academic Procrastination | 5 |
| Xu et al (2014) | 95(95) | China | _ | Lay | junior school student | General Procrastination | 3 |
| Xu et al (2016) | 284(289) | China | - | Ran Hong | Junior school student | Academic Procrastination | 5 |
| Xu et al (2016) | 304(356) | China | - | Lay | high school student | General Procrastination | 5 |
| Xu et al (2016) | 113(268) | China | 20.05 ± 1.027 | Lay | college student | General Procrastination | 4 |
| Xu et al (2018) | 178(559) | China | - | Lay | college student | General Procrastination | 4 |
| Yan et al (2013) | 302(295) | China | - | PASS | college student | Academic Procrastination | 4 |
| Yang et al (2009) | 158(153) | China | - | Lay | Primary student | General Procrastination | 4 |
| Yang et al (2014) | 160(129) | China | - | Milgram | college student | Academic Procrastination | 5 |
| Yang et al (2015) | 281(285) | China | - | PASS | graduate student | Academic Procrastination | 5 |
| Yang et al (2015) | 128(279) | China | _ | PASS | junior school student | Academic Procrastination | 4 |
| Yang et al (2019） | 275(386) | China | - | Lay | Primary student | General Procrastination | 5 |
| Yao et al (2012) | 122(227) | China | - | PASS | College student | Academic Procrastination | 4 |
| Yao et al (2015) | 181(227) | China | - | PASS | Junior school student | Academic Procrastination | 5 |
| Yao et al (2020) | 47(430) | China | 17.79 | Aitken | college student | Academic Procrastination | 4 |
| Yu et al (2017) | 231(250) | China | - | Lay | primary school student | General Procrastination | 5 |
| Yuan et al (2016) | 186(374) | China | - | Lay | College student | General Procrastination | 4 |
| Zeng et al (2019) | 242(160) | China | - | PASS | High school student | Academic Procrastination | 5 |
| Zeng et al(2015） | 361(319) | China | _ | Lay | college student | General Procrastination | 4 |
| Zhai et al (2017) | 521(462) | China | - | PASS | Junior school student | Academic Procrastination | 5 |
| Zhang et al (2012) | 222(286) | China | - | Milgram | junior school student | Academic Procrastination | 4 |
| Zhang et al (2013) | 232(246) | China | - | Lay | primary school student | General Procrastination | 5 |
| Zhang et al (2015) | 276(418) | China | - | PASS | college student | Academic Procrastination | 5 |
| Zhang et al (2016) | 174(393) | China | 21.2 ± 1.4 | Lay | college student | General Procrastination | 4 |
| Zhang et al (2017) | 146(272) | China | _ | PASS | college student | Academic Procrastination | 4 |
| Zhang et al (2018) | 366(415) | China | - | Lay | college student | General Procrastination | 5 |
| Zhang et al (2019) | 170(138) | China | - | Lay | Primary student | General Procrastination | 4 |
| Zhang et al (2019) | 178(213) | China | - | PASS | College student | Academic Procrastination | 5 |
| Zhang et al (2019) | 184(195) | China | - | PASS | College student | Academic Procrastination | 5 |
| Zhang et al (2019) | 300(279) | China | _ | Lay | college student | General Procrastination | 4 |
| Zhang et al(2013) | 136(135) | China | 23.78 | PASS | graduate student | Academic Procrastination | 4 |
| Zhang et al(2014) | 2(212) | China | _ | Lay | college student | General Procrastination | 3 |
| Zhao et al (2009) | 589(509) | China | - | PASS | college student | Academic Procrastination | 4 |
| Zhao et al (2011) | 235(209) | China | - | Zuo Yanmei | junior school student | Academic Procrastination | 5 |
| Zhao et al (2012) | 394(372) | China | - | PASS | junior school student | Academic Procrastination | 5 |
| Zhao et al (2014) | 150(436) | China | - | PASS | college student | Academic Procrastination | 3.5 |
| Zhao et al (2017) | 161(176) | China | - | Zuo Yanmei | Junior school student | Academic Procrastination | 5 |
| Zhao et al (2018) | 274(303) | China | - | Aitken | college student | Academic Procrastination | 5 |
| Zhen et al (2015) | 91(92) | China | _ | PASS | college student | Academic Procrastination | 4 |
| Zheng et al (2014) | 189(228) | China | - | PASS | college student | Academic Procrastination | 4 |
| Zhou et al (2013) | 332(355) | China | - | Aitken | junior school student | Academic Procrastination | 5 |
| Zhou et al (2016) | 61(139) | China | _ | Aitken | college student | Academic Procrastination | 3 |
| Zhou et al (2019) | 242(254) | China | - | PASS | High school student | Academic Procrastination | 5 |
| Zhou et al (2019) | 50(503) | China | - | PASS | college student | Academic Procrastination | 4 |
| Zong et al (2017) | 288(306) | China | - | Zuo Yanmei | High school student | Academic Procrastination | 5 |
| Zong et al (2020) | 419(458) | China | - | PASS | college student | Academic Procrastination | 5 |

**Table S1** Summary of included studies for meta-analysis towards gender differences of procrastination. Scores mean the final results of literature quality by using modified Newcastle-Ottawa quality assessment
