# Supplementary material for: Gender, Socioeconomic Status, Cultural Differences, Education, Family Size and Procrastination: A Sociodemographic Meta-Analysis
Source: Front Psychol. 2022 Jan 5;12:719425. doi: 10.3389/fpsyg.2021.719425 (PMC8766341; doi:10.3389/fpsyg.2021.719425)
Supplement: Supplementary file 2 [file Data_Sheet_1.doc]

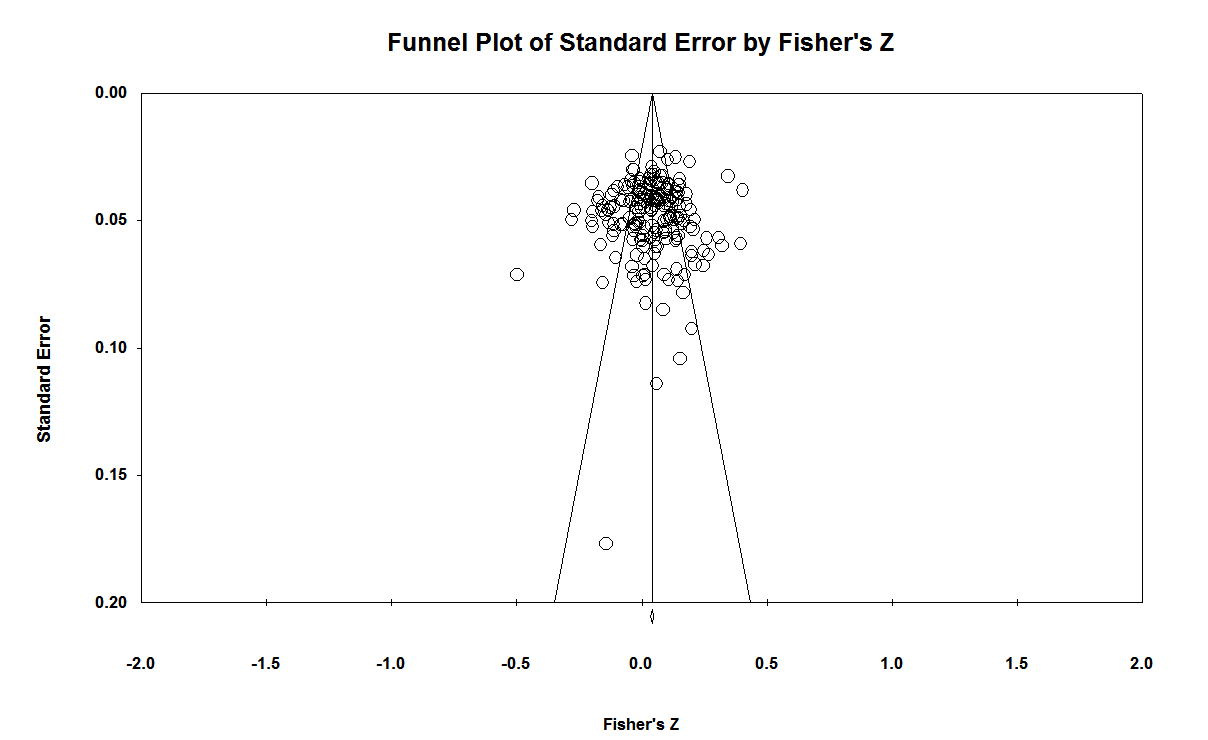
**Figure S1** Funnel plot for the meta-analytic results of gender differences of procrastination.

**Table S2** The results of modified Newcastle-Ottawa quality control assessment for meta-analysis towards gender differences of procrastination.There is a balanced gender ratio in this study (relative male/female ratio < 65 %); 2. Sample size > 300; 3. Effective response rates > 85 %; 4. This study provides details for the validation of scales used in it (e.g., Validity); 5. This study deploys well-validated and widely-used scales.

| Author | Year | Modified Newcastle-Ottawa quality assessment item | | | | | | | | | | | | | | Score 1 | Score 2 |
| --- | --- | --- | --- | --- | --- | --- | --- | --- | --- | --- | --- | --- | --- | --- | --- | --- | --- |
|  |  | 1 | | 2 | | | 3 | | | 4 | | | | 5 | |  |  |
| Ma | 2012 | √ | √ | | √ | √ | | √ | √ | | √ | √ | √ | | √ | 5 | 5 |
| Xu | 2016 | √ | √ | | √ | √ | | √ | √ | | √ | √ | √ | | √ | 5 | 5 |
| Zeng | 2019 | √ | √ | | √ | √ | | √ | √ | | √ | √ | √ | | √ | 5 | 5 |
| Sun | 2016 | √ | √ | | √ | √ | | √ | √ | | √ | √ | √ | | √ | 5 | 5 |
| Deng | 2013 | √ | √ | | √ | √ | | √ | √ | | √ | √ | √ | | √ | 5 | 5 |
| He | 2019 | √ | √ | | √ | √ | | √ | √ | | √ | √ | √ | | √ | 5 | 5 |
| Mao | 2018 | √ | √ | | √ | √ | | √ | √ | | √ | √ | √ | | √ | 5 | 5 |
| Jia | 2020 | √ | √ | | √ | √ | | √ | √ | | √ | √ | √ | | √ | 5 | 5 |
| Wei | 2016 | × | × | | √ | √ | | √ | √ | | √ | √ | √ | | √ | 4 | 4 |
| Wang | 2014 | √ | √ | | × | × | | √ | √ | | √ | √ | √ | | √ | 4 | 4 |
| Xiao | 2018 | √ | √ | | × | × | | √ | √ | | √ | √ | √ | | √ | 4 | 4 |
| Hu | 2019 | √ | √ | | √ | √ | | √ | √ | | √ | √ | √ | | √ | 5 | 5 |
| Yang | 2019 | √ | √ | | √ | √ | | √ | √ | | √ | √ | √ | | √ | 5 | 5 |
| Gao | 2017 | √ | √ | | × | × | | √ | √ | | √ | √ | √ | | √ | 4 | 4 |
| Li | 2013 | √ | √ | | √ | √ | | √ | √ | | √ | √ | √ | | √ | 5 | 5 |
| Jin | 2015 | √ | √ | | √ | √ | | √ | √ | | √ | √ | × | | × | 4 | 4 |
| Li | 2012 | √ | √ | | √ | √ | | √ | √ | | √ | √ | √ | | √ | 5 | 5 |
| Huang | 2019 | √ | √ | | × | × | | √ | √ | | √ | √ | √ | | √ | 4 | 4 |
| Yang | 2009 | √ | √ | | √ | √ | | × | × | | √ | √ | √ | | √ | 4 | 4 |
| Li | 2015 | √ | √ | | √ | √ | | √ | √ | | √ | √ | √ | | √ | 5 | 5 |
| Li | 2020 | √ | √ | | √ | √ | | √ | √ | | √ | √ | √ | | √ | 5 | 5 |
| Lv | 2018 | √ | √ | | × | × | | √ | √ | | √ | √ | √ | | √ | 4 | 4 |
| Zhang | 2019 | √ | √ | | × | × | | √ | √ | | √ | √ | √ | | √ | 4 | 4 |
| Zhao | 2017 | √ | √ | | √ | √ | | √ | √ | | √ | √ | √ | | √ | 5 | 5 |
| Jiang | 2012 | × | × | | √ | √ | | √ | √ | | √ | √ | √ | | √ | 4 | 4 |
| Yao | 2012 | × | × | | √ | √ | | √ | √ | | √ | √ | √ | | √ | 4 | 4 |
| Wen | 2014 | √ | √ | | √ | √ | | √ | √ | | √ | √ | √ | | √ | 5 | 5 |
| Li | 2019 | √ | √ | | √ | √ | | √ | √ | | √ | √ | √ | | √ | 5 | 5 |
| Li | 2014 | √ | √ | | √ | √ | | √ | √ | | √ | √ | √ | | √ | 5 | 5 |
| Shi | 2019 | √ | √ | | √ | √ | | √ | √ | | √ | √ | √ | | √ | 5 | 5 |
| Diao | 2015 | × | × | | × | × | | √ | √ | | √ | √ | √ | | √ | 3 | 3 |
| Xing | 2019 | √ | √ | | √ | √ | | √ | √ | | √ | √ | √ | | √ | 5 | 5 |
| Yao | 2015 | √ | √ | | √ | √ | | √ | √ | | √ | √ | √ | | √ | 5 | 5 |
| Zhang | 2019 | √ | √ | | √ | √ | | √ | √ | | √ | √ | √ | | √ | 5 | 5 |
| Li | 2019 | √ | √ | | √ | √ | | √ | √ | | √ | √ | √ | | √ | 5 | 5 |
| Zhang | 2019 | √ | √ | | √ | √ | | √ | √ | | √ | √ | √ | | √ | 5 | 5 |
| Ding | 2016 | √ | √ | | √ | √ | | √ | √ | | √ | √ | √ | | √ | 5 | 5 |
| Dai | 2018 | √ | √ | | √ | √ | | √ | √ | | √ | √ | √ | | √ | 5 | 5 |
| Zhai | 2017 | √ | √ | | √ | √ | | √ | √ | | √ | √ | √ | | √ | 5 | 5 |
| Li | 2019 | √ | √ | | √ | √ | | √ | √ | | √ | √ | √ | | √ | 5 | 5 |
| Xie | 2018 | × | × | | √ | √ | | √ | √ | | √ | √ | √ | | √ | 4 | 4 |
| Peng | 2009 | √ | √ | | × | × | | √ | √ | | √ | √ | √ | | √ | 4 | 4 |
| Jing | 2019 | √ | √ | | √ | √ | | √ | √ | | √ | √ | √ | | √ | 5 | 5 |
| Lu | 2017 | √ | √ | | √ | √ | | √ | √ | | √ | √ | √ | | √ | 5 | 5 |
| Tian | 2016 | √ | √ | | √ | √ | | √ | √ | | √ | √ | √ | | √ | 5 | 5 |
| Xu | 2014 | √ | √ | | √ | √ | | √ | √ | | √ | √ | √ | | √ | 5 | 5 |
| Li | 2018 | √ | √ | | √ | √ | | √ | √ | | √ | √ | √ | | √ | 5 | 5 |
| Wang | 2015 | √ | √ | | √ | √ | | √ | √ | | √ | √ | √ | | √ | 5 | 5 |
| Qu | 2019 | √ | √ | | √ | √ | | √ | √ | | √ | √ | √ | | √ | 5 | 5 |
| Zong | 2017 | √ | √ | | √ | √ | | √ | √ | | √ | √ | √ | | √ | 5 | 5 |
| Li | 2016 | √ | √ | | √ | √ | | √ | √ | | √ | √ | √ | | √ | 5 | 5 |
| Zhou | 2019 | √ | √ | | √ | √ | | √ | √ | | √ | √ | √ | | √ | 5 | 5 |
| Liu | 2011 | × | × | | × | × | | √ | √ | | √ | √ | √ | | √ | 3 | 3 |
| Gao | 2018 | √ | √ | | √ | √ | | √ | √ | | √ | √ | √ | | √ | 5 | 5 |
| Tian | 2017 | √ | √ | | √ | √ | | √ | √ | | √ | √ | √ | | √ | 5 | 5 |
| Guo | 2015 | √ | √ | | √ | √ | | √ | √ | | √ | √ | √ | | √ | 5 | 5 |
| Suo | 2015 | √ | √ | | √ | √ | | × | × | | √ | √ | √ | | √ | 4 | 4 |
| Liu | 2017 | √ | √ | | √ | √ | | √ | √ | | √ | √ | √ | | √ | 5 | 5 |
| Yuan | 2016 | × | × | | √ | √ | | √ | √ | | √ | √ | √ | | √ | 4 | 4 |
| Chen | 2019 | √ | √ | | √ | √ | | √ | √ | | √ | √ | √ | | √ | 5 | 5 |
| He | 2018 | × | × | | √ | √ | | √ | √ | | √ | √ | √ | | √ | 4 | 4 |
| Gao | 2018 | √ | √ | | √ | √ | | √ | √ | | √ | √ | √ | | √ | 5 | 5 |
| Wu | 2015 | √ | √ | | √ | √ | | √ | √ | | √ | √ | √ | | √ | 5 | 5 |
| Chen | 2009 | √ | √ | | √ | √ | | √ | √ | | √ | √ | √ | | √ | 5 | 5 |
| Ding | 2015 | √ | √ | | √ | √ | | √ | √ | | √ | √ | √ | | √ | 5 | 5 |
| Liu | 2018 | × | × | | √ | √ | | √ | √ | | √ | √ | √ | | √ | 4 | 4 |
| Zhao | 2014 | × | × | | √ | √ | | × | √ | | √ | √ | √ | | √ | 3 | 4 |
| Tong | 2017 | √ | √ | | √ | √ | | √ | √ | | √ | √ | √ | | √ | 5 | 5 |
| Liu | 2014 | √ | √ | | √ | √ | | √ | √ | | √ | √ | √ | | √ | 5 | 5 |
| Zhang | 2018 | √ | √ | | √ | √ | | √ | √ | | √ | √ | √ | | √ | 5 | 5 |
| Ni | 2015 | √ | √ | | √ | √ | | √ | √ | | √ | √ | √ | | √ | 5 | 5 |
| Xu | 2018 | × | × | | √ | √ | | √ | √ | | √ | √ | √ | | √ | 4 | 4 |
| Gao | 2015 | √ | √ | | √ | √ | | √ | √ | | √ | √ | √ | | √ | 5 | 5 |
| Yang | 2014 | √ | √ | | √ | √ | | √ | √ | | √ | √ | √ | | √ | 5 | 5 |
| Zong | 2020 | √ | √ | | √ | √ | | √ | √ | | √ | √ | √ | | √ | 5 | 5 |
| Zhang | 2015 | √ | √ | | √ | √ | | √ | √ | | √ | √ | √ | | √ | 5 | 5 |
| Zhao | 2009 | √ | √ | | √ | √ | | × | × | | √ | √ | √ | | √ | 4 | 4 |
| Zhao | 2018 | √ | √ | | √ | √ | | √ | √ | | √ | √ | √ | | √ | 5 | 5 |
| Guo | 2015 | √ | √ | | × | × | | × | × | | √ | √ | √ | | √ | 3 | 3 |
| Wang | 2009 | √ | √ | | × | × | | √ | √ | | √ | √ | √ | | √ | 4 | 4 |
| Wang | 2017 | √ | √ | | √ | √ | | √ | √ | | √ | √ | √ | | √ | 5 | 5 |
| Tian | 2018 | √ | √ | | √ | √ | | √ | √ | | √ | √ | √ | | √ | 5 | 5 |
| Zhang | 2012 | √ | √ | | √ | √ | | √ | √ | | √ | √ | × | | × | 4 | 4 |
| Ji | 2019 | √ | √ | | √ | √ | | √ | √ | | √ | √ | √ | | √ | 5 | 5 |
| Zhao | 2012 | √ | √ | | √ | √ | | √ | √ | | √ | √ | √ | | √ | 5 | 5 |
| Li | 2020 | √ | √ | | × | × | | √ | √ | | √ | √ | √ | | √ | 4 | 4 |
| Liang | 2015 | √ | √ | | √ | √ | | √ | √ | | √ | √ | √ | | √ | 5 | 5 |
| Sun | 2018 | √ | √ | | √ | √ | | √ | √ | | √ | √ | √ | | √ | 5 | 5 |
| Wang | 2012 | √ | √ | | √ | √ | | √ | √ | | √ | √ | √ | | √ | 5 | 5 |
| Jia | 2017 | √ | √ | | √ | √ | | √ | √ | | √ | √ | √ | | √ | 5 | 5 |
| Wang | 2012 | √ | √ | | √ | √ | | √ | √ | | √ | √ | × | | × | 4 | 4 |
| Gou | 2018 | √ | √ | | √ | √ | | √ | √ | | √ | √ | √ | | √ | 5 | 5 |
| Yu | 2017 | √ | √ | | √ | √ | | √ | √ | | √ | √ | √ | | √ | 5 | 5 |
| Shao | 2018 | √ | √ | | √ | √ | | √ | √ | | √ | √ | √ | | √ | 5 | 5 |
| Chen | 2020 | √ | √ | | √ | √ | | √ | √ | | √ | √ | √ | | √ | 5 | 5 |
| Wei | 2020 | √ | √ | | √ | √ | | √ | √ | | √ | √ | √ | | √ | 5 | 5 |
| Li | 2019 | √ | √ | | √ | √ | | √ | √ | | √ | √ | √ | | √ | 5 | 5 |
| Li | 2019 | √ | √ | | √ | √ | | × | × | | √ | √ | √ | | √ | 4 | 4 |
| Zhou | 2019 | × | × | | √ | √ | | √ | √ | | √ | √ | √ | | √ | 4 | 4 |
| Liu | 2020 | √ | √ | | × | × | | √ | √ | | √ | √ | √ | | √ | 4 | 4 |
| Yang | 2015 | √ | √ | | √ | √ | | √ | √ | | √ | √ | √ | | √ | 5 | 5 |
| Li | 2016 | √ | √ | | × | × | | √ | √ | | √ | √ | √ | | √ | 4 | 4 |
| Wang | 2019 | √ | √ | | √ | √ | | √ | √ | | √ | √ | √ | | √ | 5 | 5 |
| Xu | 2016 | √ | √ | | √ | √ | | √ | √ | | √ | √ | √ | | √ | 5 | 5 |
| Lai | 2015 | √ | √ | | √ | √ | | √ | √ | | √ | √ | √ | | √ | 5 | 5 |
| Xu | 2017 | √ | √ | | √ | √ | | √ | √ | | √ | √ | √ | | √ | 5 | 5 |
| Chen | 2017 | √ | √ | | √ | √ | | √ | √ | | √ | √ | √ | | √ | 5 | 5 |
| Wang | 2020 | √ | √ | | √ | √ | | √ | √ | | √ | √ | √ | | √ | 5 | 5 |
| Chang | 2020 | √ | √ | | √ | √ | | √ | √ | | √ | √ | √ | | √ | 5 | 5 |
| Li | 2011 | √ | √ | | √ | √ | | √ | √ | | √ | √ | √ | | √ | 5 | 5 |
| Wang | 2008 | √ | √ | | √ | √ | | √ | √ | | √ | √ | √ | | √ | 5 | 5 |
| Yan | 2013 | √ | √ | | × | × | | √ | √ | | √ | √ | √ | | √ | 4 | 4 |
| Shen | 2012 | √ | √ | | √ | √ | | √ | √ | | √ | √ | √ | | √ | 5 | 5 |
| Li | 2012 | × | × | | √ | √ | | √ | √ | | √ | √ | √ | | √ | 4 | 4 |
| Zhao | 2011 | √ | √ | | √ | √ | | √ | √ | | √ | √ | √ | | √ | 5 | 5 |
| Guo | 2012 | × | × | | √ | √ | | √ | √ | | √ | √ | √ | | √ | 4 | 4 |
| Tang | 2012 | √ | √ | | √ | √ | | √ | √ | | √ | √ | √ | | √ | 5 | 5 |
| Kong | 2013 | √ | √ | | √ | √ | | √ | √ | | √ | √ | √ | | √ | 5 | 5 |
| Zhang | 2013 | √ | √ | | √ | √ | | √ | √ | | √ | √ | √ | | √ | 5 | 5 |
| Qin | 2013 | √ | √ | | √ | √ | | √ | √ | | √ | √ | √ | | √ | 5 | 5 |
| Liu | 2013 | √ | √ | | √ | √ | | √ | √ | | √ | √ | √ | | √ | 5 | 5 |
| Huang | 2013 | √ | √ | | √ | √ | | √ | √ | | √ | √ | √ | | √ | 5 | 5 |
| Zhou | 2013 | √ | √ | | √ | √ | | √ | √ | | √ | √ | √ | | √ | 5 | 5 |
| Shi | 2013 | √ | √ | | √ | √ | | √ | √ | | √ | √ | √ | | √ | 5 | 5 |
| Song | 2013 | √ | √ | | × | × | | √ | √ | | √ | √ | √ | | √ | 4 | 4 |
| Fang | 2014 | √ | √ | | √ | √ | | √ | √ | | √ | √ | √ | | √ | 5 | 5 |
| Zheng | 2014 | √ | √ | | √ | √ | | × | × | | √ | √ | √ | | √ | 4 | 4 |
| Sun | 2013 | × | × | | √ | √ | | √ | √ | | √ | √ | √ | | √ | 4 | 4 |
| Kuang | 2012 | √ | √ | | √ | √ | | √ | √ | | √ | √ | √ | | √ | 5 | 5 |
| Wang | 2014 | √ | √ | | √ | √ | | √ | √ | | √ | √ | √ | | √ | 5 | 5 |
| Sun | 2014 | √ | √ | | √ | √ | | √ | √ | | × | × | √ | | √ | 4 | 4 |
| Wang | 2016 | √ | √ | | √ | √ | | √ | √ | | √ | √ | √ | | √ | 5 | 5 |
| Liu | 2013 | √ | √ | | √ | √ | | √ | √ | | √ | √ | √ | | √ | 5 | 5 |
| Li | 2017 | √ | √ | | × | × | | √ | √ | | √ | √ | √ | | √ | 4 | 4 |
| Zeng | 2015 | √ | √ | | √ | √ | | √ | √ | | √ | √ | × | | × | 4 | 4 |
| Bian | 2017 | √ | √ | | √ | √ | | √ | √ | | √ | √ | √ | | √ | 5 | 5 |
| Liang | 2012 | √ | √ | | √ | √ | | √ | √ | | √ | √ | √ | | √ | 5 | 5 |
| Shen | 2016 | × | × | | √ | √ | | √ | √ | | √ | √ | √ | | √ | 4 | 4 |
| Huang | 2015 | √ | √ | | × | × | | × | × | | × | × | √ | | √ | 2 | 2 |
| Luo | 2014 | √ | √ | | × | × | | √ | √ | | × | × | √ | | √ | 3 | 3 |
| Xu | 2016 | × | × | | √ | √ | | √ | √ | | √ | √ | √ | | √ | 4 | 4 |
| Huang | 2018 | × | × | | √ | √ | | √ | √ | | √ | √ | √ | | √ | 4 | 4 |
| Lu | 2011 | √ | √ | | √ | √ | | √ | √ | | √ | √ | √ | | √ | 5 | 5 |
| Zhang | 2014 | × | × | | × | × | | √ | √ | | √ | √ | √ | | √ | 3 | 3 |
| Qiu | 2013 | √ | √ | | √ | √ | | √ | √ | | √ | √ | √ | | √ | 5 | 5 |
| Wu | 2019 | √ | √ | | × | × | | × | × | | √ | √ | √ | | √ | 3 | 3 |
| Gao | 2017 | √ | √ | | √ | √ | | √ | √ | | √ | √ | √ | | √ | 5 | 5 |
| Yang | 2015 | √ | √ | | √ | √ | | √ | √ | | × | × | √ | | √ | 4 | 4 |
| Zhang | 2013 | √ | √ | | √ | √ | | √ | √ | | × | × | √ | | √ | 4 | 4 |
| Shao | 2017 | √ | √ | | × | × | | √ | √ | | √ | √ | √ | | √ | 4 | 4 |
| Song | 2020 | √ | √ | | √ | √ | | √ | √ | | √ | √ | √ | | √ | 5 | 5 |
| Li et | 2013 | √ | √ | | √ | √ | | √ | √ | | √ | √ | √ | | √ | 5 | 5 |
| Song | 2013 | √ | √ | | × | × | | √ | √ | | × | × | √ | | √ | 3 | 3 |
| Feng | 2020 | √ | √ | | √ | √ | | √ | √ | | √ | √ | √ | | √ | 5 | 5 |
| Hou | 2021 | √ | √ | | √ | √ | | √ | √ | | √ | √ | √ | | √ | 5 | 5 |
| Zhang | 2019 | √ | √ | | √ | √ | | √ | √ | | × | × | √ | | √ | 4 | 4 |
| Deng | 2019 | × | × | | √ | √ | | √ | √ | | × | × | √ | | √ | 3 | 3 |
| Dong | 2020 | × | × | | × | × | | √ | √ | | √ | √ | √ | | √ | 3 | 3 |
| Yao | 2020 | × | × | | √ | √ | | √ | √ | | √ | √ | √ | | √ | 4 | 4 |
| Li | 2017 | × | × | | √ | √ | | √ | √ | | √ | √ | √ | | √ | 4 | 4 |
| Jiao | 2017 | × | × | | √ | √ | | √ | √ | | × | × | √ | | √ | 3 | 3 |
| Chen | 2019 | √ | √ | | × | × | | √ | √ | | √ | √ | √ | | √ | 4 | 4 |
| Cui | 2014 | × | × | | √ | √ | | √ | √ | | √ | √ | √ | | √ | 4 | 4 |
| Ma | 2016 | √ | √ | | √ | √ | | √ | √ | | √ | √ | √ | | √ | 5 | 5 |
| Xu | 2014 | √ | √ | | × | × | | √ | √ | | × | × | √ | | √ | 3 | 3 |
| Sun | 2014 | √ | √ | | √ | √ | | √ | √ | | × | × | √ | | √ | 4 | 4 |
| Huang | 2014 | √ | √ | | √ | √ | | √ | √ | | √ | √ | √ | | √ | 5 | 5 |
| Pang | 2009 | √ | √ | | √ | √ | | √ | √ | | √ | √ | × | | × | 4 | 4 |
| Hou | 2008 | × | × | | × | × | | √ | √ | | √ | √ | √ | | √ | 3 | 3 |
| Xu | 2011 | √ | √ | | × | × | | √ | √ | | √ | √ | √ | | √ | 4 | 4 |
| Wan | 2020 | √ | √ | | √ | √ | | √ | √ | | √ | √ | √ | | √ | 5 | 5 |
| Wang | 2016 | √ | √ | | √ | √ | | √ | √ | | √ | √ | √ | | √ | 5 | 5 |
| Huang | 2017 | √ | √ | | √ | √ | | √ | √ | | × | × | √ | | √ | 4 | 4 |
| Zhang | 2017 | × | × | | √ | √ | | √ | √ | | √ | √ | √ | | √ | 4 | 4 |
| Zhen | 2015 | √ | √ | | × | × | | √ | √ | | √ | √ | √ | | √ | 4 | 4 |
| Xiao | 2010 | √ | √ | | √ | √ | | √ | √ | | √ | √ | √ | | √ | 5 | 5 |
| Zhou | 2016 | × | × | | × | × | | √ | √ | | √ | √ | √ | | √ | 3 | 3 |
| Qu | 2016 | √ | √ | | √ | √ | | √ | √ | | √ | √ | √ | | √ | 5 | 5 |
| Shan | 2016 | √ | √ | | √ | √ | | √ | √ | | √ | √ | √ | | √ | 5 | 5 |
| Zhang | 2016 | × | × | | √ | √ | | √ | √ | | √ | √ | √ | | √ | 4 | 4 |
| Aynur | 2011 | × | × | | √ | √ | | √ | √ | | √ | √ | √ | | √ | 4 | 4 |
| Nilüfer | 2017 | √ | √ | | √ | √ | | √ | √ | | √ | √ | √ | | √ | 5 | 5 |
| Joanne | 2014 | × | × | | × | × | | √ | × | | √ | √ | √ | | √ | 3 | 2 |
| Mojeed | 2007 | √ | √ | | × | × | | √ | √ | | √ | √ | √ | | √ | 4 | 4 |
| Hoora | 2012 | √ | √ | | × | × | | √ | × | | √ | √ | √ | | √ | 4 | 3 |
| Carla M | 2016 | √ | √ | | × | × | | √ | √ | | × | × | √ | | √ | 3 | 3 |
| Erkan | 2011 | √ | √ | | √ | √ | | × | × | | √ | √ | √ | | √ | 4 | 4 |
| Ruhsan | 2011 | √ | √ | | × | × | | √ | √ | | × | × | √ | | √ | 3 | 3 |
| Adel | 2013 | √ | √ | | × | × | | √ | √ | | √ | √ | √ | | √ | 4 | 4 |
| Laurel A. | 1998 | √ | √ | | × | × | | √ | √ | | √ | √ | √ | | √ | 4 | 4 |
| Richard | 2014 | √ | √ | | × | × | | × | √ | | √ | √ | √ | | √ | 3 | 4 |
| Djilali | 2018 | √ | √ | | √ | √ | | √ | √ | | √ | √ | √ | | √ | 5 | 5 |
| Ajayi | 2020 | √ | √ | | × | × | | √ | × | | √ | √ | √ | | √ | 4 | 3 |

**Table S3** Summary of included studies for meta-analysis towards socioeconomic status differences of procrastination. Scores mean the final results of literature quality by using modified Newcastle-Ottawa quality assessment

| Author (year) | Gender, male (female) | Country/Areas | Age (Mean ± S.D) | Assessments | Identity | Type | Quality scores |
| --- | --- | --- | --- | --- | --- | --- | --- |
| Xu et al (2016) | 284(289) | China | - | Ran Hong | junior school student | Academic Procrastination | 5 |
| Sun et al (2016) | 184(292) | China | - | Zuo Yanmei | high school student | Academic Procrastination | 5 |
| Mao et al (2018) | 194(207) | China | - | PASS | college students | Academic Procrastination | 5 |
| Li et al (2012) | 479(466) | China | - | Milgram | primary student | Academic Procrastination | 5 |
| Jiang et al (2012) | 212(403) | China | - | Lay | college student | General Procrastination | 4 |
| Yao et al (2012) | 122(227) | China | - | PASS | college student | Academic Procrastination | 4 |
| Xing et al (2019) | 282(279) | China | - | PASS | college student | Academic Procrastination | 5 |
| Yao et al (2015) | 181(227) | China | - | PASS | junior school student | Academic Procrastination | 5 |
| Zhang et al (2019)a | 178(213) | China | - | PASS | college student | Academic Procrastination | 5 |
| Zhang et al (2019)b | 184(195) | China | - | PASS | college student | Academic Procrastination | 5 |
| Xie et al (2018) | 385(190) | China | - | Zuo Yanmei | middle school student | Academic Procrastination | 4 |
| Liu et al (2011) | 67(131) | China | - | Lay | college student | General Procrastination | 3 |
| Guo et al (2015) | 307(274) | China | - | PASS | Junior school student | Academic Procrastination | 5 |
| Liu et al (2017) | 225(276) | China | - | PASS | college student | Academic Procrastination | 5 |
| He et al (2018) | 160(354) | China | - | Akiten | college student | Academic Procrastination | 4 |
| Gao et al (2018) | 268(241) | China | - | PASS | college student | Academic Procrastination | 5 |
| Wu et al (2015) | 175(288) | China | - | PASS | college student | Academic Procrastination | 5 |
| Tong et al (2017) | 377(363) | China | - | PASS | college student | Academic Procrastination | 5 |
| Ni et al (2015) | 200(184) | China | - | Lay | college student | General Procrastination | 5 |
| Yang et al (2015) | 281(285) | China | - | PASS | graduate student | Academic Procrastination | 5 |
| Lai et al (2015) | 466(338) | China | - | PASS | college student | Academic Procrastination | 5 |
| Wang et al (2020) | 326(481) | China | - | Akiten | college student | Academic Procrastination | 5 |
| Li et al (2012) | 113(227) | China | - | Akiten | college student | Academic Procrastination | 4 |
| Kuang et al (2012) | 330(598) | China | - | Lay | college student | General Procrastination | 5 |
| Liu et al(2013) | 421(314) | China | _ | Akiten | junior school student | Academic Procrastination | 5 |
| Bian et al(2017) | 154(192) | China | _ | Lay | college student | General Procrastination | 5 |
| Liang et al (2012) | 146(161) | China | _ | Akiten | college student | Academic Procrastination | 5 |
| Luo et al (2014) | 72(113) | China | _ | PASS | college student | Academic Procrastination | 3 |
| Hou et al (2021) | 377(500) | China | _ | PASS | college student | Academic Procrastination | 5 |
| Deng et al (2019) | 153(481) | China | 20 ± 1 | PASS | college student | Academic Procrastination | 3 |
| Dong et al (2020) | 57(164) | China | _ | PASS | college student | Academic Procrastination | 3 |
| Yao et al (2020) | 47(430) | China | 17.79 | Akiten | college student | Academic Procrastination | 4 |
| Chen et al (2019) | 108(132) | China | _ | PASS | college student | Academic Procrastination | 4 |
| Wan et al (2020) | 611(570) | China | _ | Lay | primary school student | General Procrastination | 5 |
| Huang et al (2017) | 391(549) | China | 20.54 ± 1.41 | Lay | college student | General Procrastination | 4 |
| Wang et al(2017) | 182(194) | China | _ | PASS | college student | Academic Procrastination | 5 |
| Zhou et al (2016) | 61(139) | China | _ | Akiten | college student | Academic Procrastination | 3 |
| Qu et al (2016) | 170(198) | China | _ | Akiten | college student | Academic Procrastination | 5 |
| Shan et al (2016) | 436(380) | China | 20 ± 2 | Lay | college student | General Procrastination | 5 |
| Zhang et al (2016) | 174(393) | China | 21.2 ± 1.4 | Lay | college student | General Procrastination | 4 |

**
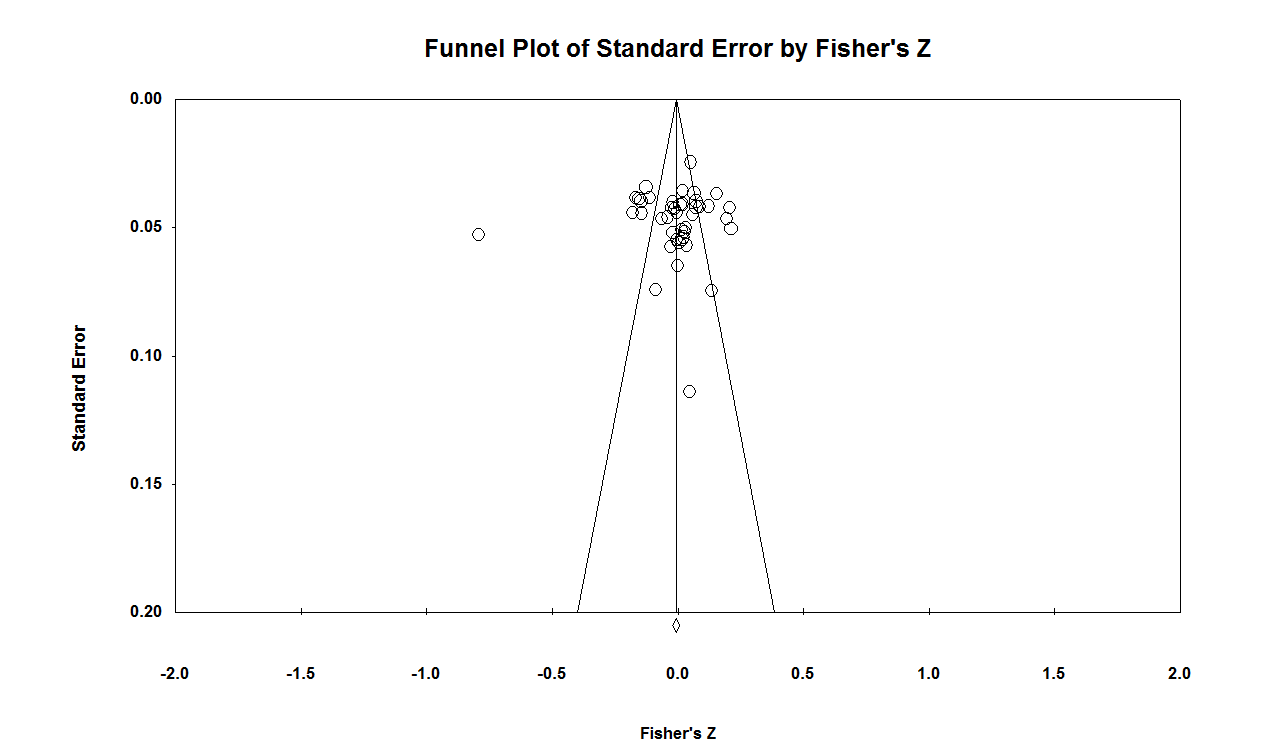
**

**Figure S2** Funnel plot for the meta-analytic results of socioeconomic status (SES) differences of procrastination.

**Table S4** The results of modified Newcastle-Ottawa quality control assessment for meta-analysis towards socioeconomic status differences of procrastination.There is a balanced gender ratio in this study (relative male/female ratio < 65 %); 2. Sample size > 300; 3. Effective response rates > 85 %; 4. This study provides details for the validation of scales used in it (e.g., Validity); 5. This study deploys well-validated and widely-used scales.

| Author | Year | Modified Newcastle-Ottawa quality assessment item | | | | | | | | | | Score 1 | Score 2 |
| --- | --- | --- | --- | --- | --- | --- | --- | --- | --- | --- | --- | --- | --- |
|  |  | 1 | | 2 | | 3 | | 4 | | 5 | |  |  |
| Xu | 2016 | √ | √ | √ | √ | √ | √ | √ | √ | √ | √ | 5 | 5 |
| Sun | 2016 | √ | √ | √ | √ | √ | √ | √ | √ | √ | √ | 5 | 5 |
| Mao | 2018 | √ | √ | √ | √ | √ | √ | √ | √ | √ | √ | 5 | 5 |
| Li | 2012 | √ | √ | √ | √ | √ | √ | √ | √ | √ | √ | 5 | 5 |
| Jiang | 2012 | × | × | √ | √ | √ | √ | √ | √ | √ | √ | 4 | 4 |
| Yao | 2012 | × | × | √ | √ | √ | √ | √ | √ | √ | √ | 4 | 4 |
| Xing | 2019 | √ | √ | √ | √ | √ | √ | √ | √ | √ | √ | 5 | 5 |
| Yao | 2015 | √ | √ | √ | √ | √ | √ | √ | √ | √ | √ | 5 | 5 |
| Zhang | 2019 | √ | √ | √ | √ | √ | √ | √ | √ | √ | √ | 5 | 5 |
| Zhang | 2019 | √ | √ | √ | √ | √ | √ | √ | √ | √ | √ | 5 | 5 |
| Xie | 2018 | × | × | √ | √ | √ | √ | √ | √ | √ | √ | 4 | 4 |
| Liu | 2011 | × | × | × | × | √ | √ | √ | √ | √ | √ | 3 | 3 |
| Guo | 2015 | √ | √ | √ | √ | √ | √ | √ | √ | √ | √ | 5 | 5 |
| Liu | 2017 | √ | √ | √ | √ | √ | √ | √ | √ | √ | √ | 5 | 5 |
| He | 2018 | × | × | √ | √ | √ | √ | √ | √ | √ | √ | 4 | 4 |
| Gao | 2018 | √ | √ | √ | √ | √ | √ | √ | √ | √ | √ | 5 | 5 |
| Wu | 2015 | √ | √ | √ | √ | √ | √ | √ | √ | √ | √ | 5 | 5 |
| Tong | 2017 | √ | √ | √ | √ | √ | √ | √ | √ | √ | √ | 5 | 5 |
| Ni | 2015 | √ | √ | √ | √ | √ | √ | √ | √ | √ | √ | 5 | 5 |
| Yang | 2015 | √ | √ | √ | √ | √ | √ | √ | √ | √ | √ | 5 | 5 |
| Lai | 2015 | √ | √ | √ | √ | √ | √ | √ | √ | √ | √ | 5 | 5 |
| Wang | 2020 | √ | √ | √ | √ | √ | √ | √ | √ | √ | √ | 5 | 5 |
| Li | 2012 | × | × | √ | √ | √ | √ | √ | √ | √ | √ | 4 | 4 |
| Kuang | 2012 | √ | √ | √ | √ | √ | √ | √ | √ | √ | √ | 5 | 5 |
| Liu | 2013 | √ | √ | √ | √ | √ | √ | √ | √ | √ | √ | 5 | 5 |
| Bian | 2017 | √ | √ | √ | √ | √ | √ | √ | √ | √ | √ | 5 | 5 |
| Liang | 2012 | √ | √ | √ | √ | √ | √ | √ | √ | √ | √ | 5 | 5 |
| Luo | 2014 | √ | √ | × | × | √ | √ | × | × | √ | √ | 3 | 3 |
| Hou | 2021 | √ | √ | √ | √ | √ | √ | √ | √ | √ | √ | 5 | 5 |
| Deng | 2019 | × | × | √ | √ | √ | √ | × | × | √ | √ | 3 | 3 |
| Dong | 2020 | × | × | × | × | √ | √ | √ | √ | √ | √ | 3 | 3 |
| Yao | 2020 | × | × | √ | √ | √ | √ | √ | √ | √ | √ | 4 | 4 |
| Chen | 2019 | √ | √ | × | × | √ | √ | √ | √ | √ | √ | 4 | 4 |
| Wan | 2020 | √ | √ | √ | √ | √ | √ | √ | √ | √ | √ | 5 | 5 |
| Huang | 2017 | √ | √ | √ | √ | √ | √ | × | × | √ | √ | 4 | 4 |
| Wang | 2017 | √ | √ | √ | √ | √ | √ | √ | √ | √ | √ | 5 | 5 |
| Zhou | 2016 | × | × | × | × | √ | √ | √ | √ | √ | √ | 3 | 3 |
| Qu | 2016 | √ | √ | √ | √ | √ | √ | √ | √ | √ | √ | 5 | 5 |
| Shan | 2016 | √ | √ | √ | √ | √ | √ | √ | √ | √ | √ | 5 | 5 |
| Zhang | 2016 | × | × | √ | √ | √ | √ | √ | √ | √ | √ | 4 | 4 |

**Table S5** Summary of included studies for meta-analysis towards multicultural status differences of procrastination. Scores mean the final results of literature quality by using modified Newcastle-Ottawa quality assessment

| Author (year) | Gender, male (female) | Country/Areas | Age (Mean ± S.D) | Assessments | Identity | Type | Quality scores |
| --- | --- | --- | --- | --- | --- | --- | --- |
| Song et al (2014) | 200(488) | China | - | PASS | college student | Academic Procrastination | 4 |
| Kuang et al (2012) | 330(598) | China | - | Lay | college student | General Procrastination | 5 |
| Wang et al (2014) | 206(290) | China | - | PASS | college student | Academic Procrastination | 5 |
| Chen et al (2019) | 108(132) | China | _ | PASS | college student | Academic Procrastination | 4 |
| Cui et al (2014) | 29(143) | China | _ | PASS | college student | Academic Procrastination | 4 |
| Zhang et al (2016) | 174(393) | China | 21.2 ± 1.4 | Lay | college student | General Procrastination | 4 |

**
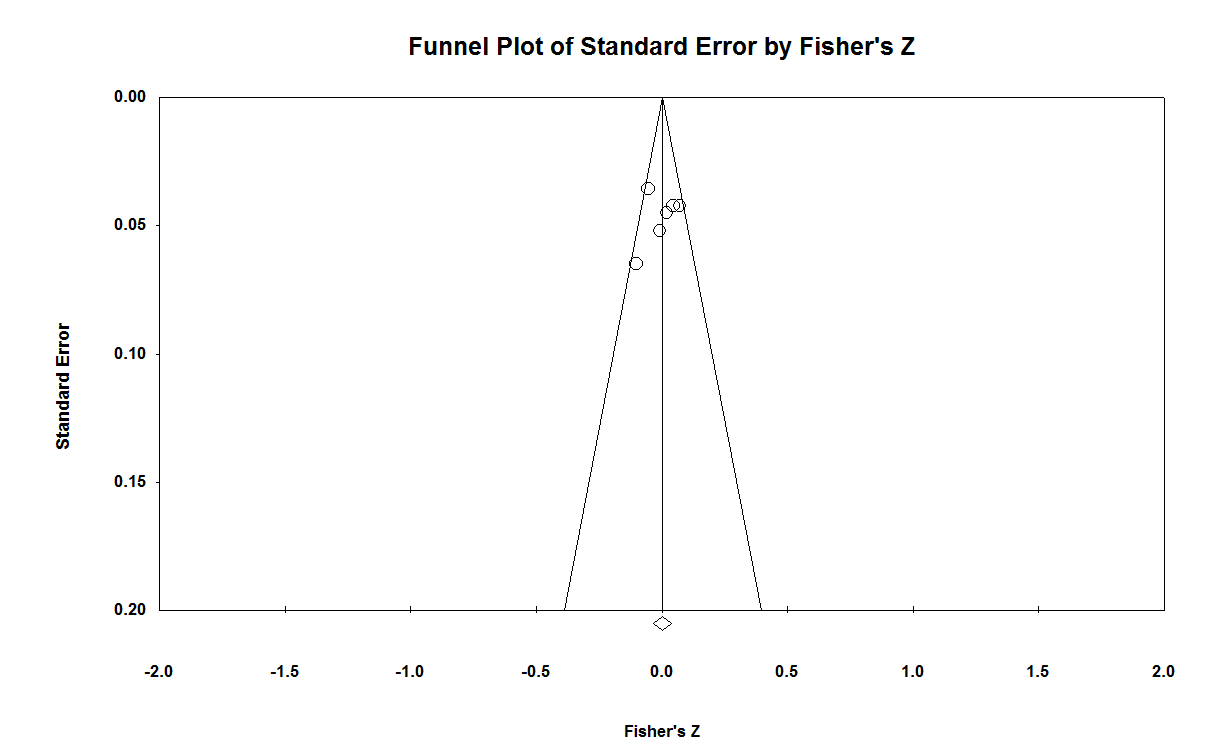
**

**Figure S3** Funnel plot for the meta-analytic results of multicultural differences of procrastination.

| Author | Year | Modified Newcastle-Ottawa quality assessment item | | | | | | | | | | Score 1 | Score 2 |
| --- | --- | --- | --- | --- | --- | --- | --- | --- | --- | --- | --- | --- | --- |
|  |  | 1 | | 2 | | 3 | | 4 | | 5 | |  |  |
| Song | 2014 | √ | √ | × | × | √ | √ | √ | √ | √ | √ | 4 | 4 |
| Kuang | 2012 | √ | √ | √ | √ | √ | √ | √ | √ | √ | √ | 5 | 5 |
| Wang | 2014 | √ | √ | √ | √ | √ | √ | √ | √ | √ | √ | 5 | 5 |
| Chen | 2019 | √ | √ | × | × | √ | √ | √ | √ | √ | √ | 4 | 4 |
| Cui | 2014 | × | × | √ | √ | √ | √ | √ | √ | √ | √ | 4 | 4 |
| Zhang | 2016 | × | × | √ | √ | √ | √ | √ | √ | √ | √ | 4 | 4 |

**Table S6** The results of modified Newcastle-Ottawa quality control assessment for meta-analysis towards multicultural differences of procrastination.There is a balanced gender ratio in this study (relative male/female ratio < 65 %); 2. Sample size > 300; 3. Effective response rates > 85 %; 4. This study provides details for the validation of scales used in it (e.g., Validity); 5. This study deploys well-validated and widely-used scales.

**Table S7** Summary of included studies for meta-analysis towards family size differences of procrastination. Scores mean the final results of literature quality by using modified Newcastle-Ottawa quality assessment.

| Author (year) | Gender, male (female) | Country/Areas | Age (Mean ± S.D) | Assessments | Identity | Type | Quality scores |
| --- | --- | --- | --- | --- | --- | --- | --- |
| Xu et al (2016) | 284(289) | China | - | Ran Hong | junior school student | Academic Procrastination | 5 |
| Sun et al (2016) | 184(292) | China | - | Zuo Yanmei | high school student | Academic Procrastination | 5 |
| Yang et al (2019) | 275(386) | China | - | Lay | primary school student | General Procrastination | 5 |
| Gao et al (2017) | 150(104) | China | - | Lay | primary school student | General Procrastination | 4 |
| Li et al (2012) | 479(466) | China | - | Milgram | primary school student | Academic Procrastination | 5 |
| Huang et al (2019) | 116(96) | China | - | Milgram | primary school student | Academic Procrastination | 4 |
| Yang et al (2009) | 158(153) | China | - | Lay | primary school student | General Procrastination | 4 |
| Li et al (2020) | 330(281) | China | - | Lay | primary school student | General Procrastination | 5 |
| Zhang et al (2019) | 170(138) | China | - | Lay | primary school student | General Procrastination | 4 |
| Zhao et al (2017) | 161(176) | China | - | Zuo Yanmei | junior school student | Academic Procrastination | 5 |
| Li et al (2013) | 250(375) | China | - | PASS | graduate student | Academic Procrastination | 5 |
| Xing et al (2019) | 282(279) | China | - | PASS | college student | Academic Procrastination | 5 |
| Yao et al (2015) | 181(227) | China | - | PASS | junior school student | Academic Procrastination | 5 |
| Zhang et al (2019) | 178(213) | China | - | PASS | college student | Academic Procrastination | 5 |
| Zhang et al (2019) | 184(195) | China | - | PASS | college student | Academic Procrastination | 5 |
| Lu et al (2017) | 315(252) | China | 16.2 ± 0.9 | Zuo Yanmei | high school student | Academic Procrastination | 5 |
| Li et al (2016) | 193(309) | China | - | Akiten | college student | Academic Procrastination | 5 |
| Liu et al (2011) | 67(131) | China | - | Lay | college student | General Procrastination | 3 |
| Gao et al (2018) | 174(176) | China | - | Lay | primary school student | General Procrastination | 5 |
| Yuan et al (2016) | 186(374) | China | - | Lay | college student | General Procrastination | 4 |
| Chen et al (2019) | 294(391) | China | - | PASS | college student | Academic Procrastination | 5 |
| He et al (2018) | 160(354) | China | - | Akiten | college student | Academic Procrastination | 4 |
| Wu et al (2015) | 175(288) | China | - | PASS | college student | Academic Procrastination | 5 |
| Tong et al (2017) | 377(363) | China | - | PASS | college student | Academic Procrastination | 5 |
| Zhang et al (2018) | 366(415) | China | - | Lay | college student | General Procrastination | 5 |
| Zhao et al (2018) | 274(303) | China | - | Akiten | college student | Academic Procrastination | 5 |
| Wang et al (2017) | 476(474) | China | - | Ran Hong | junior school student | Academic Procrastination | 5 |
| Wang et al (2012) | 285(269) | China | - | Milgram | primary school student | Academic Procrastination | 5 |
| Gou et al (2018) | 235(185) | China | - | Lay | primary school student | General Procrastination | 5 |
| Shao et al (2018) | 203(271) | China | - | Akiten | high school student | Academic Procrastination | 5 |
| Zhou et al (2019) | 50(503) | China | - | PASS | college student | Academic Procrastination | 4 |
| Yang et al (2015) | 281(285) | China | - | PASS | graduate student | Academic Procrastination | 5 |
| Lai et al (2015) | 466(338) | China | - | PASS | college student | Academic Procrastination | 5 |
| Wang et al (2020) | 326(481) | China | - | Akiten | college student | Academic Procrastination | 5 |
| Chang et al (2020) | 119(203) | China | - | Lay | primary school student | General Procrastination | 5 |
| Yan et al (2013) | 302(295) | China | - | PASS | college student | Academic Procrastination | 4 |
| Li et al (2012) | 113(227) | China | - | Akiten | college student | Academic Procrastination | 4 |
| Zhao et al (2011) | 235(209) | China | - | Zuo Yanmei | junior school student | Academic Procrastination | 5 |
| Guo et al (2012) | 280(124) | China | - | Lay | graduate student | General Procrastination | 4 |
| Qin et al (2013) | 316(235) | China | - | PASS | college student | Academic Procrastination | 5 |
| Zhou et al (2013) | 332(355) | China | - | Akiten | junior school student | Academic Procrastination | 5 |
| Song et al (2014) | 200(488) | China | - | PASS | college student | Academic Procrastination | 4 |
| Zheng et al (2014) | 189(228) | China | - | PASS | college student | Academic Procrastination | 4 |
| Sun et al (2013) | 230(244) | China | - | PASS | junior school student | Academic Procrastination | 4 |
| Kuang et al (2012) | 330(598) | China | - | Lay | college student | General Procrastination | 5 |
| Liang et al (2012) | 146(161) | China | - | Akiten | college student | Academic Procrastination | 5 |
| Shao et al (2017) | 133(147) | China | - | Akiten | college student | Academic Procrastination | 5 |
| Song et al (2013) | 147(234) | China | - | PASS | college student | Academic Procrastination | 3 |
| Hou et al (2021) | 377(500) | China | - | PASS | college student | Academic Procrastination | 5 |
| Deng et al (2019) | 153(481) | China | 20 ± 1 | PASS | college student | Academic Procrastination | 3 |
| Yao et al (2020) | 47(430) | China | 17.79 | Akiten | college student | Academic Procrastination | 4 |
| Li et al (2017) | 194(420) | China | - | PASS | college student | Academic Procrastination | 4 |
| Chen et al (2019) | 108(132) | China | - | PASS | college student | Academic Procrastination | 4 |
| Xu et al (2014) | 95(95) | China | - | Lay | junior school student | General Procrastination | 3 |
| Wan et al (2020) | 611(570) | China | - | Lay | primary school student | General Procrastination | 5 |
| Huang et al (2017) | 391(549) | China | 20.54 ± 1.41 | Lay | college student | General Procrastination | 4 |
| Xiao et al (2010） | 385(407) | China | 20.94 ± 2.47 | Lay | college student | General Procrastination | 5 |
| Zhou et al (2016) | 61(139) | China | - | Akiten | college student | Academic Procrastination | 3 |
| Qu et al (2016) | 170(198) | China | - | Akiten | college student | Academic Procrastination | 5 |
| Zhang et al (2016) | 174(393) | China | 21.2 ± 1.4 | Lay | college student | General Procrastination | 4 |
| Wang et al (2015) | 241 (176) | China | - | PASS | college student | Academic Procrastination | 4 |


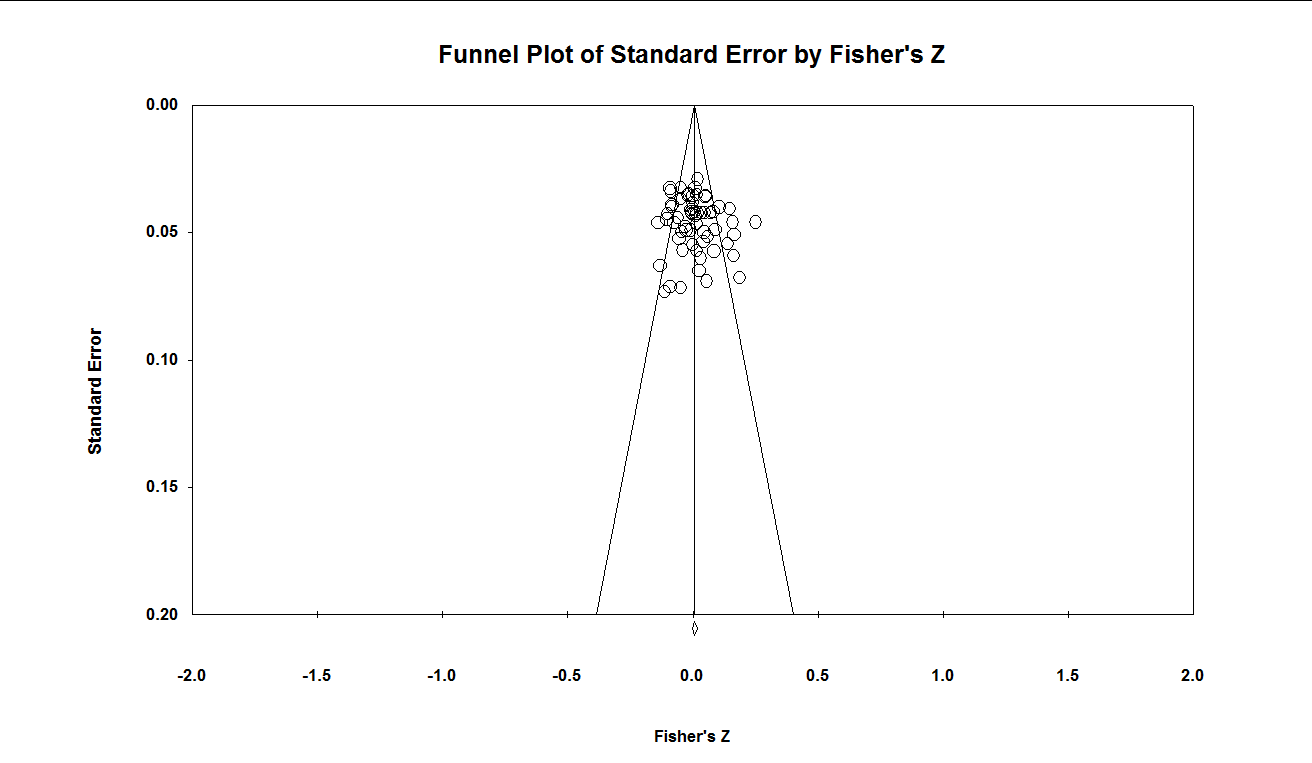
**Figure S4** Funnel plot for the meta-analytic results of family size differences of procrastination.

**Table S8** The results of modified Newcastle-Ottawa quality control assessment for meta-analysis towards family size differences of procrastination.There is a balanced gender ratio in this study (relative male/female ratio < 65 %); 2. Sample size > 300; 3. Effective response rates > 85 %; 4. This study provides details for the validation of scales used in it (e.g., Validity); 5. This study deploys well-validated and widely-used scales.

| Author | Year | Modified Newcastle-Ottawa quality assessment item | | | | | | | | | | Score 1 | Score 2 |
| --- | --- | --- | --- | --- | --- | --- | --- | --- | --- | --- | --- | --- | --- |
|  |  | 1 | | 2 | | 3 | | 4 | | 5 | |  |  |
| Xu | 2016 | √ | √ | √ | √ | √ | √ | √ | √ | √ | √ | 5 | 5 |
| Sun | 2016 | √ | √ | √ | √ | √ | √ | √ | √ | √ | √ | 5 | 5 |
| Yang | 2019 | √ | √ | √ | √ | √ | √ | √ | √ | √ | √ | 5 | 5 |
| Gao | 2017 | √ | √ | × | × | √ | √ | √ | √ | √ | √ | 4 | 4 |
| Li | 2012 | √ | √ | √ | √ | √ | √ | √ | √ | √ | √ | 5 | 5 |
| Huang | 2019 | √ | √ | × | × | √ | √ | √ | √ | √ | √ | 4 | 4 |
| Yang | 2009 | √ | √ | √ | √ | × | × | √ | √ | √ | √ | 4 | 4 |
| Li | 2020 | √ | √ | √ | √ | √ | √ | √ | √ | √ | √ | 5 | 5 |
| Zhang | 2019 | √ | √ | √ | √ | × | × | √ | √ | √ | √ | 4 | 4 |
| Zhao | 2017 | √ | √ | √ | √ | √ | √ | √ | √ | √ | √ | 5 | 5 |
| Li | 2013 | √ | √ | √ | √ | √ | √ | √ | √ | √ | √ | 5 | 5 |
| Xing | 2019 | √ | √ | √ | √ | √ | √ | √ | √ | √ | √ | 5 | 5 |
| Yao | 2015 | √ | √ | √ | √ | √ | √ | √ | √ | √ | √ | 5 | 5 |
| Zhang | 2019 | √ | √ | √ | √ | × | × | √ | √ | √ | √ | 5 | 5 |
| Zhang | 2019 | √ | √ | √ | √ | √ | √ | √ | √ | √ | √ | 5 | 5 |
| Lu | 2017 | √ | √ | √ | √ | √ | √ | √ | √ | √ | √ | 5 | 5 |
| Li | 2016 | √ | √ | √ | √ | √ | √ | √ | √ | √ | √ | 5 | 5 |
| Liu | 2011 | × | × | × | × | √ | √ | √ | √ | √ | √ | 3 | 3 |
| Gao | 2018 | √ | √ | √ | √ | √ | √ | √ | √ | √ | √ | 5 | 5 |
| Yuan | 2016 | × | × | √ | √ | √ | √ | √ | √ | √ | √ | 4 | 4 |
| Chen | 2019 | √ | √ | √ | √ | √ | √ | √ | √ | √ | √ | 5 | 5 |
| He | 2018 | × | × | √ | √ | √ | √ | √ | √ | √ | √ | 4 | 4 |
| Wu | 2015 | √ | √ | √ | √ | √ | √ | √ | √ | √ | √ | 5 | 5 |
| Tong | 2017 | √ | √ | √ | √ | √ | √ | √ | √ | √ | √ | 5 | 5 |
| Zhang | 2018 | √ | √ | √ | √ | √ | √ | √ | √ | √ | √ | 5 | 5 |
| Zhao | 2018 | √ | √ | √ | √ | √ | √ | √ | √ | √ | √ | 5 | 5 |
| Wang | 2017 | √ | √ | √ | √ | √ | √ | √ | √ | √ | √ | 5 | 5 |
| Wang | 2012 | √ | √ | √ | √ | √ | √ | √ | √ | √ | √ | 5 | 5 |
| Gou | 2018 | √ | √ | √ | √ | √ | √ | √ | √ | √ | √ | 5 | 5 |
| Shao | 2018 | √ | √ | √ | √ | √ | √ | √ | √ | √ | √ | 5 | 5 |
| Zhou | 2019 | × | × | √ | √ | √ | √ | √ | √ | √ | √ | 4 | 4 |
| Yang | 2015 | √ | √ | √ | √ | √ | √ | √ | √ | √ | √ | 5 | 5 |
| Lai | 2015 | √ | √ | √ | √ | √ | √ | √ | √ | √ | √ | 5 | 5 |
| Wang | 2020 | √ | √ | √ | √ | √ | √ | √ | √ | √ | √ | 5 | 5 |
| Chang | 2020 | √ | √ | √ | √ | √ | √ | √ | √ | √ | √ | 5 | 5 |
| Yan | 2013 | √ | √ | × | × | √ | √ | √ | √ | √ | √ | 4 | 4 |
| Li | 2012 | × | × | √ | √ | √ | √ | √ | √ | √ | √ | 4 | 4 |
| Zhao | 2011 | √ | √ | √ | √ | √ | √ | √ | √ | √ | √ | 5 | 5 |
| Guo | 2012 | × | × | √ | √ | √ | √ | √ | √ | √ | √ | 4 | 4 |
| Qin | 2013 | √ | √ | √ | √ | √ | √ | √ | √ | √ | √ | 5 | 5 |
| Zhou | 2013 | √ | √ | √ | √ | √ | √ | √ | √ | √ | √ | 5 | 5 |
| Song | 2014 | × | × | √ | √ | √ | √ | √ | √ | √ | √ | 4 | 4 |
| Zheng | 2014 | √ | √ | √ | √ | × | × | √ | √ | √ | √ | 4 | 4 |
| Sun | 2013 | × | × | √ | √ | √ | √ | √ | √ | √ | √ | 4 | 4 |
| Kuang | 2012 | √ | √ | √ | √ | √ | √ | √ | √ | √ | √ | 5 | 5 |
| Liang | 2012 | √ | √ | √ | √ | √ | √ | √ | √ | √ | √ | 5 | 5 |
| Shao | 2017 | √ | √ | √ | √ | √ | √ | √ | √ | √ | √ | 5 | 5 |
| Song | 2013 | √ | √ | × | × | √ | √ | × | × | √ | √ | 3 | 3 |
| Hou | 2021 | √ | √ | √ | √ | √ | √ | √ | √ | √ | √ | 5 | 5 |
| Deng | 2019 | × | × | √ | √ | √ | √ | × | × | √ | √ | 3 | 3 |
| Yao | 2020 | × | × | √ | √ | √ | √ | √ | √ | √ | √ | 4 | 4 |
| Li | 2017 | × | × | √ | √ | √ | √ | √ | √ | √ | √ | 4 | 4 |
| Chen | 2019 | √ | √ | × | × | √ | √ | √ | √ | √ | √ | 4 | 4 |
| Xu | 2014 | √ | √ | × | × | √ | √ | × | × | √ | √ | 3 | 3 |
| Wan | 2020 | √ | √ | √ | √ | √ | √ | √ | √ | √ | √ | 5 | 5 |
| Huang | 2017 | √ | √ | √ | √ | √ | √ | × | × | √ | √ | 4 | 4 |
| Xiao | 2010 | √ | √ | √ | √ | √ | √ | √ | √ | √ | √ | 5 | 5 |
| Zhou | 2016 | × | × | × | × | √ | √ | √ | √ | √ | √ | 3 | 3 |
| Qu | 2016 | √ | √ | √ | √ | √ | √ | √ | √ | √ | √ | 5 | 5 |
| Zhang | 2016 | × | × | √ | √ | √ | √ | √ | √ | √ | √ | 4 | 4 |
| Wang | 2015 | √ | √ | √ | √ | × | × | √ | √ | √ | √ | 4 | 4 |

**Table S9** Summary of included studies for meta-analysis towards educational background differences of procrastination. Scores mean the final results of literature quality by using modified Newcastle-Ottawa quality assessment.

| Author (year) | Gender, male (female) | Country/Areas | Age (Mean ± S.D) | Assessments | Identity | Type | Quality scores |
| --- | --- | --- | --- | --- | --- | --- | --- |
| Sun et al (2016) | 184(292) | China | - | Zuo Yanmei | High school student | Academic Procrastination | 5 |
| He et al (2019) | 271(332) | China | 15.32 ± 2.51 | Aitken | Middle school student | Academic Procrastination | 5 |
| Jiang et al (2012) | 212(403) | China | - | Lay | College student | General Procrastination | 4 |
| Yao et al (2012) | 122(227) | China | - | PASS | College student | Academic Procrastination | 4 |
| Li et al (2013) | 250(375) | China | - | PASS | Graduate student | Academic Procrastination | 5 |
| Xing et al (2019) | 282(279) | China | - | PASS | College student | Academic Procrastination | 5 |
| Zhang et al (2019)a | 178(213) | China | - | PASS | College student | Academic Procrastination | 5 |
| Zhang et al (2019)b | 184(195) | China | - | PASS | College student | Academic Procrastination | 5 |
| Li et al (2008) | 183(215) | China | - | PASS | High school student | Academic Procrastination | 5 |
| Suo et al (2015) | 337(519) | China | - | PASS | College student | Academic Procrastination | 4 |
| Liu et al (2017) | 225(276) | China | - | PASS | College student | Academic Procrastination | 5 |
| Chen et al (2019) | 294(391) | China | 19.4 | PASS | College student | Academic Procrastination | 5 |
| He et al (2018) | 160(354) | China | - | Akiten | College student | Academic Procrastination | 4 |
| Gao et al (2018) | 268(241) | China | - | PASS | College student | Academic Procrastination | 5 |
| Wu et al (2015) | 175(288) | China | - | PASS | College student | Academic Procrastination | 5 |
| Chen et al (2009) | 277(361) | China | - | PASS | college student | Academic Procrastination | 5 |
| Zhao et al (2014) | 150(436) | China | - | PASS | college student | Academic Procrastination | 3.5 |
| Tong et al (2017) | 377(363) | China | - | PASS | college student | Academic Procrastination | 5 |
| Gao et al (2015) | 219(322) | China | - | PASS | college student | Academic Procrastination | 5 |
| Yang et al (2014) | 160(129) | China | - | Milgram | college student | Academic Procrastination | 5 |
| Zhao et al (2018) | 274(303) | China | - | Akiten | college student | Academic Procrastination | 5 |
| Jia et al (2017) | 185(325) | China | - | PASS | graduate student | Academic Procrastination | 5 |
| Yang et al (2015) | 281(285) | China | - | PASS | graduate student | Academic Procrastination | 5 |
| Chen et al (2017) | 182(182) | China | - | PASS | college student | Academic Procrastination | 5 |
| Yan et al (2013) | 302(295) | China | - | PASS | college student | Academic Procrastination | 4 |
| Li et al (2012) | 113(227) | China | - | Akiten | college student | Academic Procrastination | 4 |
| Guo et al (2012) | 280(124) | China | - | Lay | graduate student | General Procrastination | 4 |
| Kong et al (2013) | 201(263) | China | - | PASS | college student | Academic Procrastination | 5 |
| Qin et al (2013) | 316(235) | China | - | PASS | college student | Academic Procrastination | 5 |
| Liu et al (2013) | 365(386) | China | - | Lay | college student | General Procrastination | 5 |
| Liang et al (2012) | 146(161) | China | - | Akiten | college student | Academic Procrastination | 5 |
| Luo et al (2014) | 72(113) | China | - | PASS | college student | Academic Procrastination | 3 |
| Lu et al (2011) | 163(175) | China | - | Lay | college student | General Procrastination | 5 |
| Qiu et al (2013) | 385(408) | China | - | Ran Hong | high school student | Academic Procrastination | 5 |
| Zhang et al (2019) | 300(279) | China | - | Lay | college student | General Procrastination | 4 |
| Chen et al (2019) | 108(132) | China | - | PASS | college student | Academic Procrastination | 4 |
| Cui et al (2014) | 29(143) | China | - | PASS | college student | Academic Procrastination | 4 |
| Huang et al (2014) | 200(488) | China | - | Tuckman | college student | Academic Procrastination | 5 |
| Pang et al (2009) | 742(882) | China | 20.26 | PASS | college student | Academic Procrastination | 4 |
| Wang et al (2016) | 169(143) | China | 21 ± 3 | Lay | college student | General Procrastination | 5 |
| Zhen et al (2015) | 91(92) | China | - | PASS | college student | Academic Procrastination | 4 |
| Adel et al (2013) | 40(40) | Egypt | 19.1 ± 9.5 | Akiten | college student | Academic Procrastination | 4 |


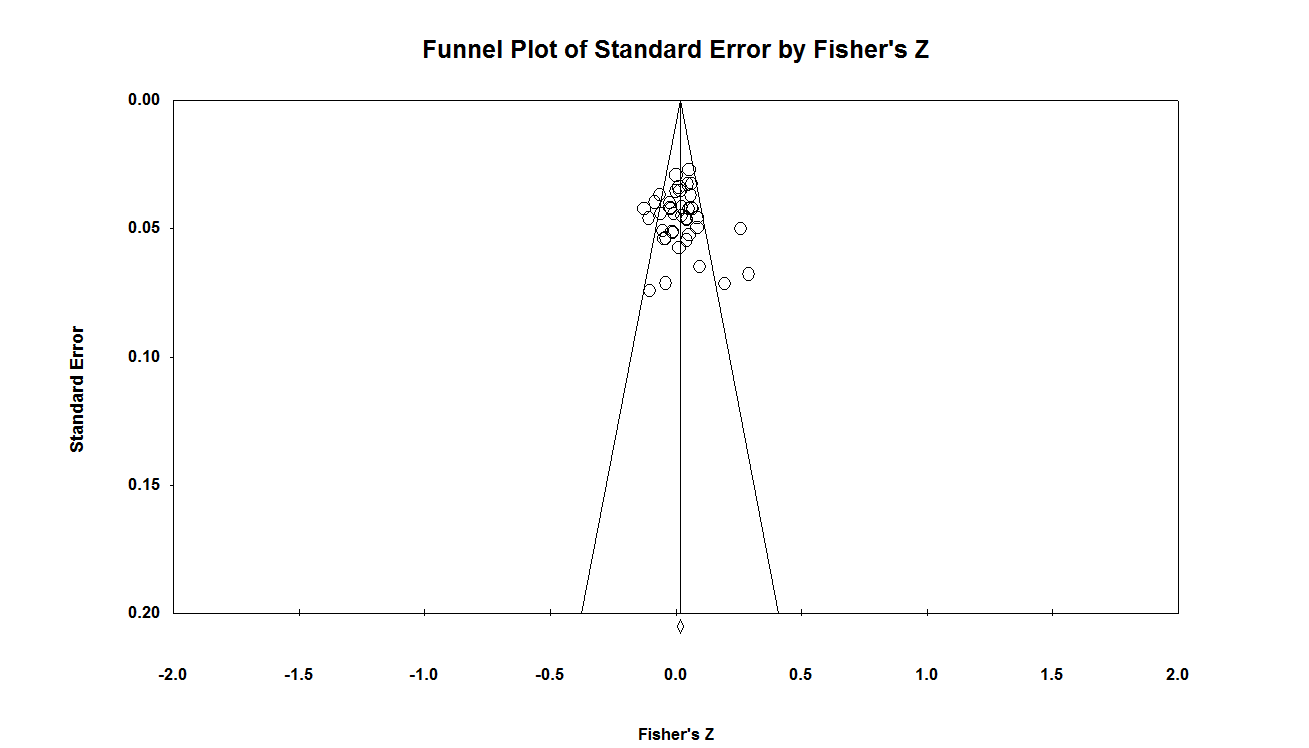
**Figure S5** Funnel plot for the meta-analytic results of educational background difference differences of procrastination.

**Table S10** The results of modified Newcastle-Ottawa quality control assessment for meta-analysis towards educational background differences of procrastination.There is a balanced gender ratio in this study (relative male/female ratio < 65 %); 2. Sample size > 300; 3. Effective response rates > 85 %; 4. This study provides details for the validation of scales used in it (e.g., Validity); 5. This study deploys well-validated and widely-used scales.

| Author | Year | Modified Newcastle-Ottawa quality assessment item | | | | | | | | | | Score 1 | Score 2 |
| --- | --- | --- | --- | --- | --- | --- | --- | --- | --- | --- | --- | --- | --- |
|  |  | 1 | | 2 | | 3 | | 4 | | 5 | |  |  |
| Sun | 2016 | √ | √ | √ | √ | √ | √ | √ | √ | √ | √ | 5 | 5 |
| He | 2019 | √ | √ | √ | √ | √ | √ | √ | √ | √ | √ | 5 | 5 |
| Jiang | 2012 | × | × | √ | √ | √ | √ | √ | √ | √ | √ | 4 | 4 |
| Yao | 2012 | × | × | √ | √ | √ | √ | √ | √ | √ | √ | 4 | 4 |
| Li | 2013 | √ | √ | √ | √ | √ | √ | √ | √ | √ | √ | 5 | 5 |
| Shi | 2019 | √ | √ | √ | √ | √ | √ | √ | √ | √ | √ | 5 | 5 |
| Xing | 2019 | √ | √ | √ | √ | √ | √ | √ | √ | √ | √ | 5 | 5 |
| Zhang | 2019 | √ | √ | √ | √ | √ | √ | √ | √ | √ | √ | 5 | 5 |
| Zhang | 2019 | √ | √ | √ | √ | √ | √ | √ | √ | √ | √ | 5 | 5 |
| Li | 2008 | √ | √ | √ | √ | √ | √ | √ | √ | √ | √ | 5 | 5 |
| Suo | 2015 | √ | √ | √ | √ | × | × | √ | √ | √ | √ | 4 | 4 |
| Liu | 2017 | √ | √ | √ | √ | √ | √ | √ | √ | √ | √ | 5 | 5 |
| Chen | 2019 | √ | √ | √ | √ | √ | √ | √ | √ | √ | √ | 5 | 5 |
| He | 2018 | × | × | √ | √ | √ | √ | √ | √ | √ | √ | 4 | 4 |
| Gao | 2018 | √ | √ | √ | √ | √ | √ | √ | √ | √ | √ | 5 | 5 |
| Wu | 2015 | √ | √ | √ | √ | √ | √ | √ | √ | √ | √ | 5 | 5 |
| Chen | 2009 | √ | √ | √ | √ | √ | √ | √ | √ | √ | √ | 5 | 5 |
| Zhao | 2014 | × | × | √ | √ | × | √ | √ | √ | √ | √ | 3 | 4 |
| Tong | 2017 | √ | √ | √ | √ | √ | √ | √ | √ | √ | √ | 5 | 5 |
| Gao | 2015 | √ | √ | √ | √ | √ | √ | √ | √ | √ | √ | 5 | 5 |
| Yang | 2014 | √ | √ | √ | √ | √ | √ | √ | √ | √ | √ | 5 | 5 |
| Zhao | 2018 | √ | √ | √ | √ | √ | √ | √ | √ | √ | √ | 5 | 5 |
| Jia | 2017 | √ | √ | √ | √ | √ | √ | √ | √ | √ | √ | 5 | 5 |
| Yang | 2015 | √ | √ | √ | √ | √ | √ | √ | √ | √ | √ | 5 | 5 |
| Chen | 2017 | √ | √ | √ | √ | √ | √ | √ | √ | √ | √ | 5 | 5 |
| Yan | 2013 | √ | √ | × | × | √ | √ | √ | √ | √ | √ | 4 | 4 |
| Li | 2012 | × | × | √ | √ | √ | √ | √ | √ | √ | √ | 4 | 4 |
| Guo | 2012 | × | × | √ | √ | √ | √ | √ | √ | √ | √ | 4 | 4 |
| Kong | 2013 | √ | √ | √ | √ | √ | √ | √ | √ | √ | √ | 5 | 5 |
| Qin | 2013 | √ | √ | √ | √ | √ | √ | √ | √ | √ | √ | 5 | 5 |
| Liu | 2013 | √ | √ | √ | √ | √ | √ | √ | √ | √ | √ | 5 | 5 |
| Liang | 2012 | √ | √ | √ | √ | √ | √ | √ | √ | √ | √ | 5 | 5 |
| Luo | 2014 | √ | √ | × | × | √ | √ | × | × | √ | √ | 3 | 3 |
| Lu | 2011 | √ | √ | √ | √ | √ | √ | √ | √ | √ | √ | 5 | 5 |
| Qiu | 2013 | √ | √ | √ | √ | √ | √ | √ | √ | √ | √ | 5 | 5 |
| Zhang | 2019 | √ | √ | √ | √ | √ | √ | × | × | √ | √ | 4 | 4 |
| Chen | 2019 | √ | √ | × | × | √ | √ | √ | √ | √ | √ | 4 | 4 |
| Cui | 2014 | × | × | √ | √ | √ | √ | √ | √ | √ | √ | 4 | 4 |
| Huang | 2014 | √ | √ | √ | √ | √ | √ | √ | √ | √ | √ | 5 | 5 |
| Pang | 2009 | √ | √ | √ | √ | √ | √ | √ | √ | × | × | 4 | 4 |
| Wang | 2016 | √ | √ | √ | √ | √ | √ | √ | √ | √ | √ | 5 | 5 |
| Zhen | 2015 | √ | √ | × | × | √ | √ | √ | √ | √ | √ | 4 | 4 |
| Adel | 2013 | √ | √ | × | × | √ | √ | √ | √ | √ | √ | 4 | 4 |

**Table S11** Jackknife analysis results. “√” means the results were re-confirmed even though this study was removed.

| Author | Year | Check |
| --- | --- | --- |
|  |  |  |
| Ma | 2012 | √ |
| Xu | 2016 | √ |
| Zeng | 2019 | √ |
| Sun | 2016 | √ |
| Deng | 2013 | √ |
| He | 2019 | √ |
| Mao | 2018 | √ |
| Jia | 2020 | √ |
| Wei | 2016 | √ |
| Wang | 2014 | √ |
| Xiao | 2018 | √ |
| Hu | 2019 | √ |
| Yang | 2019 | √ |
| Gao | 2017 | √ |
| Li | 2013 | √ |
| Jin | 2015 | √ |
| Li | 2012 | √ |
| Huang | 2019 | √ |
| Yang | 2009 | √ |
| Li | 2015 | √ |
| Li | 2020 | √ |
| Lv | 2018 | √ |
| Zhang | 2019 | √ |
| Zhao | 2017 | √ |
| Jiang | 2012 | √ |
| Yao | 2012 | √ |
| Wen | 2014 | √ |
| Li | 2019 | √ |
| Li | 2014 | √ |
| Shi | 2019 | √ |
| Diao | 2015 | √ |
| Xing | 2019 | √ |
| Yao | 2015 | √ |
| Zhang | 2019 | √ |
| Li | 2019 | √ |
| Zhang | 2019 | √ |
| Ding | 2016 | √ |
| Dai | 2018 | √ |
| Zhai | 2017 | √ |
| Li | 2019 | √ |
| Xie | 2018 | √ |
| Peng | 2009 | √ |
| Jing | 2019 | √ |
| Lu | 2017 | √ |
| Tian | 2016 | √ |
| Xu | 2014 | √ |
| Li | 2018 | √ |
| Wang | 2015 | √ |
| Qu | 2019 | √ |
| Zong | 2017 | √ |
| Li | 2016 | √ |
| Zhou | 2019 | √ |
| Liu | 2011 | √ |
| Gao | 2018 | √ |
| Tian | 2017 | √ |
| Guo | 2015 | √ |
| Suo | 2015 | √ |
| Liu | 2017 | √ |
| Yuan | 2016 | √ |
| Chen | 2019 | √ |
| He | 2018 | √ |
| Gao | 2018 | √ |
| Wu | 2015 | √ |
| Chen | 2009 | √ |
| Ding | 2015 | √ |
| Liu | 2018 | √ |
| Zhao | 2014 | √ |
| Tong | 2017 | √ |
| Liu | 2014 | √ |
| Zhang | 2018 | √ |
| Ni | 2015 | √ |
| Xu | 2018 | √ |
| Gao | 2015 | √ |
| Yang | 2014 | √ |
| Zong | 2020 | √ |
| Zhang | 2015 | √ |
| Zhao | 2009 | √ |
| Zhao | 2018 | √ |
| Guo | 2015 | √ |
| Wang | 2009 | √ |
| Wang | 2017 | √ |
| Tian | 2018 | √ |
| Zhang | 2012 | × |
| Ji | 2019 | √ |
| Zhao | 2012 | √ |
| Li | 2020 | √ |
| Liang | 2015 | √ |
| Sun | 2018 | √ |
| Wang | 2012 | √ |
| Jia | 2017 | √ |
| Wang | 2012 | √ |
| Gou | 2018 | √ |
| Yu | 2017 | √ |
| Shao | 2018 | √ |
| Chen | 2020 | √ |
| Wei | 2020 | √ |
| Li | 2019 | √ |
| Li | 2019 | √ |
| Zhou | 2019 | √ |
| Liu | 2020 | √ |
| Yang | 2015 | √ |
| Li | 2016 | √ |
| Wang | 2019 | √ |
| Xu | 2016 | √ |
| Lai | 2015 | √ |
| Xu | 2017 | √ |
| Chen | 2017 | √ |
| Wang | 2020 | √ |
| Chang | 2020 | √ |
| Li | 2011 | √ |
| Wang | 2008 | √ |
| Yan | 2013 | √ |
| Shen | 2012 | √ |
| Li | 2012 | √ |
| Zhao | 2011 | √ |
| Guo | 2012 | √ |
| Tang | 2012 | √ |
| Kong | 2013 | √ |
| Zhang | 2013 | √ |
| Qin | 2013 | √ |
| Liu | 2013 | √ |
| Huang | 2013 | √ |
| Zhou | 2013 | √ |
| Shi | 2013 | √ |
| Song | 2013 | √ |
| Fang | 2014 | √ |
| Zheng | 2014 | √ |
| Sun | 2013 | √ |
| Kuang | 2012 | √ |
| Wang | 2014 | √ |
| Sun | 2014 | √ |
| Wang | 2016 | √ |
| Liu | 2013 | √ |
| Li | 2017 | √ |
| Zeng | 2015 | √ |
| Bian | 2017 | √ |
| Liang | 2012 | √ |
| Shen | 2016 | √ |
| Huang | 2015 | √ |
| Luo | 2014 | √ |
| Xu | 2016 | √ |
| Huang | 2018 | √ |
| Lu | 2011 | √ |
| Zhang | 2014 | √ |
| Qiu | 2013 | √ |
| Wu | 2019 | √ |
| Gao | 2017 | √ |
| Yang | 2015 | √ |
| Zhang | 2013 | √ |
| Shao | 2017 | √ |
| Song | 2020 | √ |
| Li et | 2013 | √ |
| Song | 2013 | √ |
| Feng | 2020 | √ |
| Hou | 2021 | √ |
| Zhang | 2019 | √ |
| Deng | 2019 | √ |
| Dong | 2020 | √ |
| Yao | 2020 | √ |
| Li | 2017 | √ |
| Jiao | 2017 | √ |
| Chen | 2019 | √ |
| Cui | 2014 | √ |
| Ma | 2016 | √ |
| Xu | 2014 | √ |
| Sun | 2014 | √ |
| Huang | 2014 | √ |
| Pang | 2009 | × |
| Hou | 2008 | √ |
| Xu | 2011 | √ |
| Wan | 2020 | √ |
| Wang | 2016 | √ |
| Huang | 2017 | √ |
| Zhang | 2017 | √ |
| Zhen | 2015 | √ |
| Xiao | 2010 | √ |
| Zhou | 2016 | √ |
| Qu | 2016 | √ |
| Shan | 2016 | √ |
| Zhang | 2016 | √ |
| Aynur | 2011 | √ |
| Nilüfer | 2017 | √ |
| Joanne | 2014 | √ |
| Mojeed | 2007 | √ |
| Hoora | 2012 | √ |
| Carla M | 2016 | √ |
| Erkan | 2011 | √ |
| Ruhsan | 2011 | √ |
| Adel | 2013 | √ |
| Laurel A. | 1998 | √ |
| Richard | 2014 | √ |
| Djilali | 2018 | √ |
| Ajayi | 2020 | √ |
